# Supplementary material for: Efficient reduction-oxidation coupling degradation of nitroaromatic compounds in continuous flow processes
Source: Nat Commun. 2024 Jul 29;15:6364. doi: 10.1038/s41467-024-50238-8 (PMC11286756; doi:10.1038/s41467-024-50238-8)
Supplement: Supplementary file 1 — Supplementary Information [file 41467_2024_50238_MOESM1_ESM.pdf]

Efficient Reduction-Oxidation Coupling Degradation of Nitroaromatic Compounds in Continuous Flow Processes

Yueshuang Mao[1,2,3], Bingnan Yu[1,3], Pengfei Wang[1], Shuai Yue[1], Sihui Zhan[1*]

Affiliations

[1]Key Laboratory of Pollution Processes and Environmental Criteria (Ministry of Education), College of Environmental Science and Engineering, Nankai University, Tianjin, China.

[2]College of Resources and Environment Science, Shanxi University, Taiyuan, China

[3]These authors contributed equally: Yueshuang Mao, Bingnan Yu.

Corresponding author

Correspondence to: Sihui Zhan (sihuizhan@nankai.edu.cn)

Table of Contents:

1. **Supplementary Methods 1 to 7**
2. **Supplementary Figures 1 to 45**
3. **Supplementary Tables 1 to 11**
4. **Supplementary Notes 1 to 4**

1. Supplementary Methods

1.1 Materials.

Unless otherwise specified, all chemicals and reagents are of analytical grade and can be used without further purification. Lanthanum nitrate hexahydrate ($La(NO_3)_3 \cdot 6H_2O$, 99%), iron nitrate nonahydrate ($Fe(NO_3)_3 \cdot 9H_2O$, 99%), Copper nitrate trihydrate ($Cu \cdot (NO_3)_2 \cdot 3H_2O$, 99%), citric acid monohydrate ($C_6H_8O_7 \cdot H_2O$, 99%), o-nitrophenol (ONP, 99%), p-nitrophenol (PNP, 99%), o-nitrotoluene (ONT, 99%), p-nitrotoluene (PNT, 99%), o-aminophenol (OAP, 99%), tert-butanol (TBA, 99%), potassium dichromate ($K_2Cr_2O_7$, 99%) superoxide dismutase (SOD, 99%), β-carotene (99%), methanol (MeOH, 95%), sodium chloride (NaCl, 99%), sodium bicarbonate ($NaHCO_3$, 99%), humic acid (HA, 70%), sodium sulfate (Na_2SO_4, 99%), sodium acetate (CH_3COONa, 99%), peroxymonosulfate (PMS, $KHSO_5 \cdot 0.5KHSO_4 \cdot 0.5K_2SO_4$, ≥47%), 5,5-dimethyl-1-pyrroline-N-oxide (DMPO, 97%), and 4-hydroxy-2,2,6,6-tetramethyl-piperidine (TEMP, ≥98%) were purchased from Aladdin.

1.2 Characterizations

The phase and crystallinity were characterized by a Rigaku D/Max 2200PC X-ray diffractometer with Cu Kα radiation (λ = 0.15418 nm). The morphology and microstructure were inspected via Transmission Electron Microscopy (TEM, Talos f200x) equipped with energy dispersive spectroscopy (EDS). Scanning electron microscopy (SEM) images were measured by a Hitachi SU8010. X-ray photoelectron spectroscopy (XPS) and Ultraviolet photoelectron spectroscopy (UPS) were performed on a Thermal ESCALAB 250 electron spectrometer. UV-Vis diffuse reflectance spectra (DRS) were recorded on a UV-3600i Plus UV-Vis spectrophotometer (Shimadzu). N_2 adsorption-desorption isotherms were recorded with Autosorb-IQ Automated Gas Sorption Analyzer (Quantachrome). The leakage of ions was quantified by ICP-MS (X7 Series, Thermo Electron Corporation, USA). Raman spectra were collected on Horiba LabRAM HR Evolution. Fourier

transform infrared (FT-IR) spectra were recorded on a Bruker Vertex 70 FTIR spectrometer using the KBr pellet technique. The steady-state photoluminescence (PL) spectra and time-resolved photoluminescence (TRPL) spectra were collected on a fluorescence spectrometer (Edinburgh Instruments FLS980). The surface photovoltage (SPV) spectra were obtained on a CEL-SPS1000 surface photovoltage spectrometer (CEAULIGHT, Inc). Fe *L*-edge X-ray absorption spectra (XAS) were collected at BL14W1 station in Beijing Synchrotron Radiation Facility (SSRF). Si (111) double-crystal monochromator was used for data collection and an ionization chamber with a maximum current of 250 mA was used for photon detection at room temperature. ATHENA program carried out in the IFEFFIT software packages was used for data analysis. For zero field cooled (ZFC) and field cooled (FC) measurements, vacuum-dried samples were demagnetized at 293 K by setting an initial field of 300 kOe and decreasing the field stepwise to zero by oscillating at 200 Oe s^{-1}. Samples were cooled down to 2 K via a cryocooler-based cooling system at zero fields. Then an external field of 300 Oe was applied and the samples were heated to 300 K at 1 K min^{-1} and again cooled down to 2 K at 1 K min^{-1} in the 300 Oe field. The magnetization M was measured by vibrating the samples at 40 Hz. One data point was delivered for M measured within 1 s (averaging time). Hystereses measurements were carried out at 20 K and 300 K respectively for each sample by cycling the applied field from -40000 to 40000 Oe at a rate of 5 Oe/s. We further obtain the unpaired d-electron number (n) of Fe ion (eqs. 1 ~ 2)[1].

$$\mu_{eff} = \sqrt{\frac{3k}{N\mu_B^2}}\sqrt{T\chi} \approx 2.82787\sqrt{T\chi} \qquad (1)$$

$$\mu_{eff} = \sqrt{n(n+2)} \qquad (2)$$

where k is the Boltzmann constant, N is the Avogadro constant, μ_B is the Bohr magneton, χ is magnetic susceptibility, μ_{eff} is the effective magnetic moment.

The structure changes of ONP were tested by in situ UV-Visible (UV-Vis) spectrophotometry (UV-3200, Mapada, China). The concentration of ONP/OAP were analyzed by high-performance liquid chromatography

(HPLC), (SPD-20A, Shimadzu Corporation, Japan) using a 60:40 methanol-water mixture as solvent, and 278/234 nm wavelength. Degradation intermediates were analyzed by ultra performance liquid chromatography-mass spectrometry (UPLC-MS, Orbitrap Fusion, Thermo, USA). The mineralization rate was detected by total organic carbon (TOC, Analytikjena multi N/C analyzer). The in situ attenuated total reflection-Fourier transform infrared spectroscopy (ATR-FTIR) spectra data were obtained by NICOLET iS10. In situ Raman spectra were tested with an HR Evolution Raman spectrophotometer (HORIBA Scientific Inc.). In situ irradiated X-ray photoelectron spectroscopy (ISI-XPS) was carried out on an electron spectrometer (ESCALAB 210, VG, UK) to research the electron density changes on photocatalysts under light irradiation. Electron paramagnetic resonance (EPR, Bruker A300 spectrometer) spectra were performed at room temperature for determination of active species. 5 mM 5,5-dimethyl-1-pyrroline-N-oxide (DMPO) in methanol and water was used as •OH/$SO_4^{•-}$ and •O_2^- trapper, and with 5 mM 4-Amino-2,2,6,6-tetramethylpiperidine (TEMP) in water was used as $^1O_2$ trapper. Then, under irradiation by 350 W Xe lamp, the test data at 0 min and 5 min were recorded, respectively.

The vertical axis units 'a.u.' represent atomic units, and 'arb. units' represent arbitrary units.

1.3 Density function theory (DFT) calculations.

Spin-polarized first-principle calculations were performed by the DFT using the Vienna Ab-initio Simulation Package (VASP 5.4)[2]. The generalized gradient approximation (GGA) with the Perdew-Burke-Ernzerhof (PBE) functional was used to describe the electronic exchange and correlation effects[3]. Uniform G-centered k-points meshes with a resolution of $2\pi \times 0.04$ Å^{-1} and Methfessel-Paxton electronic smearing were adopted for the integration in the Brillouin zone for geometric optimization[4]. The simulation was run with a cutoff energy of 500 eV throughout the computations. These settings ensure convergence of the total energies to within 1 meV per atom. Structure relaxation proceeded until all forces on atoms were less than 1 meV Å^{-1} and the total stress

tensor was within 0.01 GPa of the target value. We carry out the calculation of GGA + U by setting the effective parameter U to explain the on-site Coulomb interaction between Fe-3d and Cu-3d electrons with U = 3 eV[5]. Atomic coordinates of the optimized computational models data in Supplementary Data 1.

The Fukui function calculation of the ONP molecule was carried out with the Gaussian 16 software[6]. The B3LYP functional and 6-31G(*) basis set was adopted for all calculations. Orbital energy level analysis was performed by Multiwfn software (Multiwfn_3.8_dev_bin_Win64)[7]. The visualization of the orbitals Fukui function was achieved using Gauss View software (GaussView 6.0.16)[8].

1.4 Electrochemical experiments.

Electrochemical analyses are carried out using an electrochemical workstation (CHI760E Instruments) with a conventional three-electrode system in 0.5 M Na_2SO_4 solution. The working photoanodes are fabricated by depositing the sample slurries (4 mg sample, 1 mL ethanol, and 20 mL Nafion) on an ITO glass (2.75 cm^2, then dried at ambient temperature and heated at 60 °C for 8 h. The counter electrode and the reference electrode are an Ag/AgCl film and a saturated calomel electrode, respectively. The measured potential vs. Ag/AgCl is converted to the reversible hydrogen electrode (RHE) scale using the Nernst equation: $E_{RHE} = E_{Ag/AgCl} + 0.197 + 0.059$ pH. Every measurement was performed on a freshly prepared electrode.

Cyclic voltammetry (CV) and Linear sweep voltammetry (LSV)measurement is conducted between −0.5 ~ +1.5 V vs. Ag/AgCl at a scan rate of 50 mV s^{-1}. Electrochemical impedance spectra (EIS) are recorded at −0.3 V vs. Ag/AgCl within a frequency range from 10^5 to 10^{-1} Hz using an AC voltage at a 5-mV amplitude. A 350W Xenon lamp (CEL-HX F300, Beijing, China) with a 420 nm and 780 nm cutoff filter was adopted for all the transient photocurrent response (TPC) tests.

The charge carrier density in the space charge region was measured in a 0.5 M Na_2SO_4 solution at

a frequency of 1 kHz in the dark, and calculated according to the eq. 3:

$$N_d=\left(\frac{2}{e\varepsilon_0\varepsilon}\right)\left[\frac{d(E_s)}{d(\frac{1}{C^2})}\right] \quad (3)$$

where $e = 1.6 \times 10^{-19}$ C, $\varepsilon_0 = 8.86 \times 10^{-12}$ F m^{-1}, $\varepsilon = 8.9$, C is the space charge capacitance in the semiconductor, ε_0 is the permittivity of the vacuum, ε is the relative permittivity of the semiconductor, E_s is the applied potential

1.5 Energy cost analysis

To demonstrate the potential application of the LFCO/PMS/Vis process for ONP degradation in wastewater substrates, an energy cost analysis was performed using the EE/O concept. EE/O is defined as the electrical energy required to achieve primary removal. In LFCO/PMS/Vis process, EE/O includes the sum of LFCO material preparation input cost (EE/O_M) and PMS input cost (Oxidant/O), which can be calculated by the following eqs. 4 ~ 5[9].

$$EE/O_M = \frac{(P\cdot t)/100}{V\cdot \lg(\frac{C_i}{C_t})} \quad (4)$$

$$Oxidant/O = \frac{C_{oxidant}}{\lg(\frac{C_i}{C_t})} \quad (5)$$

where P is the energy input of the tubular furnace and the Xenon lamp (kWh h^{-1}), t is the operation time (h), V is the reactor volume (L), C_i and C_t are the initial and the final concentrations of ONP, respectively, and $C_{oxidant}$ is the concentration of PMS (g L^{-1}). Additionally, it was assumed that 20 g of LFCO was prepared, which can be used for 100 sets of reactions. The PMS was 9.42×10^{-3} kWh g^{-1} when converting to the energy-based units. The value of C_i/C_t is 100.

1.6 Design of large-scale plant

We choose FRB practical application due to it can increase the contact area between pollutants and catalysts and improve the degradation rate. At the same time, the catalyst can be ensured to stay relatively stable in the reaction bed layer, which is not easy to lose and damage, and the service life of the FBR can reach 8-10 years,

which can be effectively applied to the wastewater containing special pollutants[10]. 1:50 equal scale amplification was used to achieve real large-scale application with industrial significance. Specific design parameters are as follows: For part 2: the outer contour of the container was cylindrical with the diameter of 8 m and the height of 4.5 m; For part 4: the outer contour of the reactor was cylindrical with the diameter of 12.5 m and the height of 4.5 m, the diameter and height of the center light source (1326 W, calculation see below) is 4 m and 4 m, respectively. The 6 cylindrical FBRs are uniformly distributed outside the light source with the diameter of 4 m and the height of 4 m. The amount of catalyst covered in each packed reactor is 2 kg, which is replaced every month. We set the six reactors to rotate at a constant speed to allow sufficient light to further increase the reaction rate. The peristaltic pump in the plant is set to 20 kW.

1.7 Cost calculation

The plant operating costs in our designed plant is calculated using eq. 6:

$$C_i = \frac{M_i + CFC + AC + E}{Q_{fi}} \tag{6}$$

where C_i is the operating cost, M_i is the materials cost, CFC is the carbon fiber felt cost, AC is the administration cost, E is the electric charge and Q_{fi} is the sewage treatment capacity/month.

M_i: the purchase cost of chemicals for synthesizing 12 kg catalyst needs to spend 1,800 CNY;

CFC: The carbon felt needs to be replaced once a month, the carbon felt 9,600 CNY once a month[10-12];

AC: the management expenses are 20,000 CNY/month;

Q_{fi}: According to the experiments results, the processing capacity of our system is 5 t/h, and the monthly processing capacity is 3600 t/month.

E includes peristaltic pump and light source with flat industrial electricity cost (0.425 CNY/kWh).

To be specific, the average light intensity accepted by the FBR reactor should be 500 ~ 800 mW cm^{-2} [2,15]. Herein, the average light intensity of 6 reactors (Radius = 2 m) are fixed at 1000 mW cm^{-2} to ensure the high-

quality effluent. However, the distribution of photons along the thickness of the reactor is non-uniform according to Helmholtz equation[16]. Therefore, the actual light intensity I(y) can be calculated according to the eq. 7:

$$\bar{I} = \frac{2\cdot\int_0^{\frac{1}{2}} I(y)\cdot dy}{L} \tag{7}$$

I(y) = light intensity profile as a function of photoreactor thickness, W m^{-2}

L = photoreactor thickness, cm.

Then, the radiant power of light source should be 1326 W according to the eq. 8:

$$I(y) = \frac{\Phi}{4\pi y^2} \tag{8}$$

Φ = radiant power (W) emitted in all directions by a radiant energy source.

y = length from the center of the light source

Here, we adopted 9-hours operation/day and operated for a month (30 days). Thus, E is 18,000 CNY/month. According to the above data, LFCO@CFC FBR cost a total of 49,400 CNY/month with the monthly wastewater treatment scale of 3,600 tons, Therefore, the cost is calculated to about 13.72 CNY/ton, which is much lower than market price (30 ~ 60 CNY/ton). All other reference materials are carried out in this device model. Due to the same parameter Settings adopted by the device, the related carbon felt costs, electricity costs and management costs are roughly the same. The main difference are the monthly sewage treatment scale and the catalyst material costs of the system device. Similarly, under the same conditions, different catalysts have different degradation rates of pollutants, resulting in the water flux and operation scale of the device are different, which mean the different treatment costs, and the same different operation scale will also bring differences in electricity usage.

2. Supplementary Figures

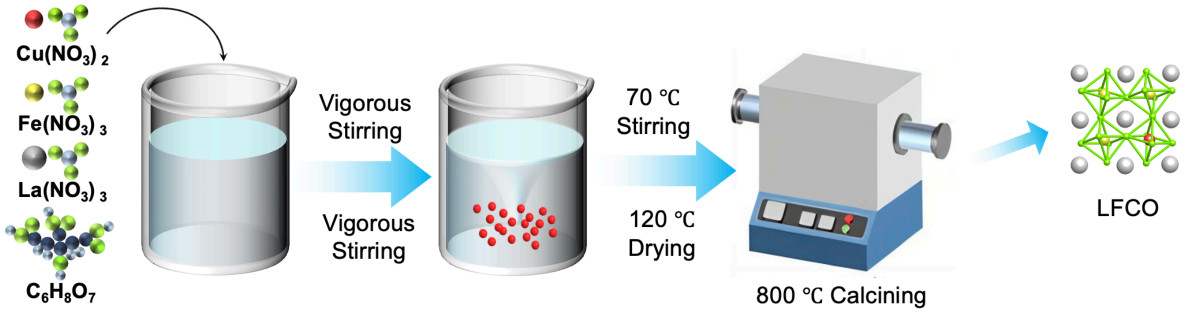

Supplementary Fig. 1 | Material synthesis diagram. The illustration of fabrication of LFCO catalyst.

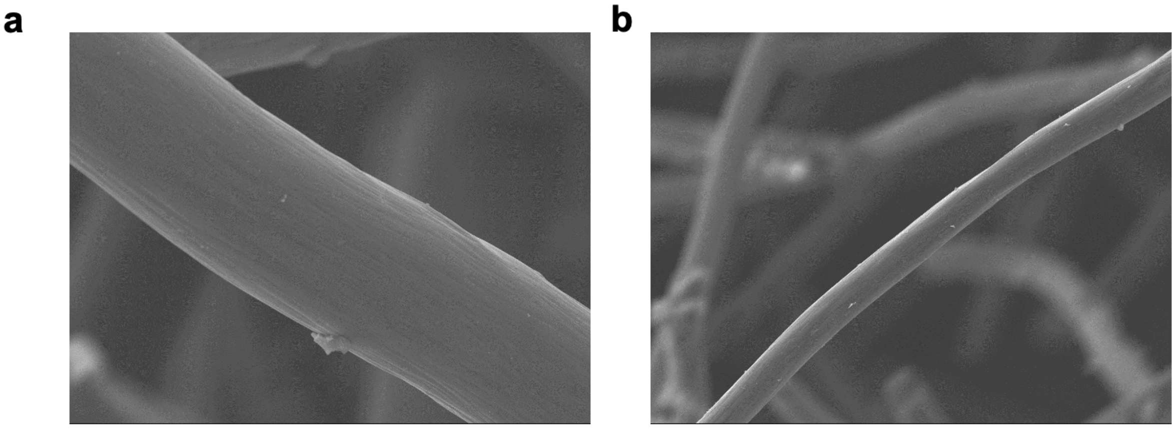

Supplementary Fig. 2 | Characterizations of CFC. (a,b) SEM images of cleaned CFC.

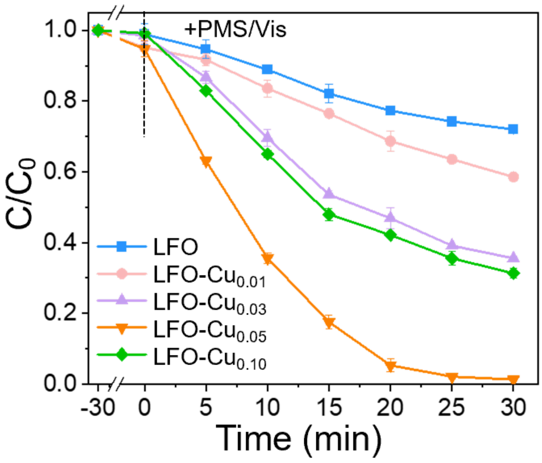

Supplementary Fig. 3 | Pre-experiments of different catalysts in beaker. Degradation of ONP in $LaFe_{1-x}Cu_xO_3$ (x = 0, 0.01, 0.03, 0.05, 0.10) in PMS system under visible light irradiation (λ > 420 nm, 50 mL 30 ppm ONP solution including 0.3 mM PMS and 0.4 g L^{-1} catalyst, initial pH = 7.1). Error bars are standard error values of three tests (n = 3).

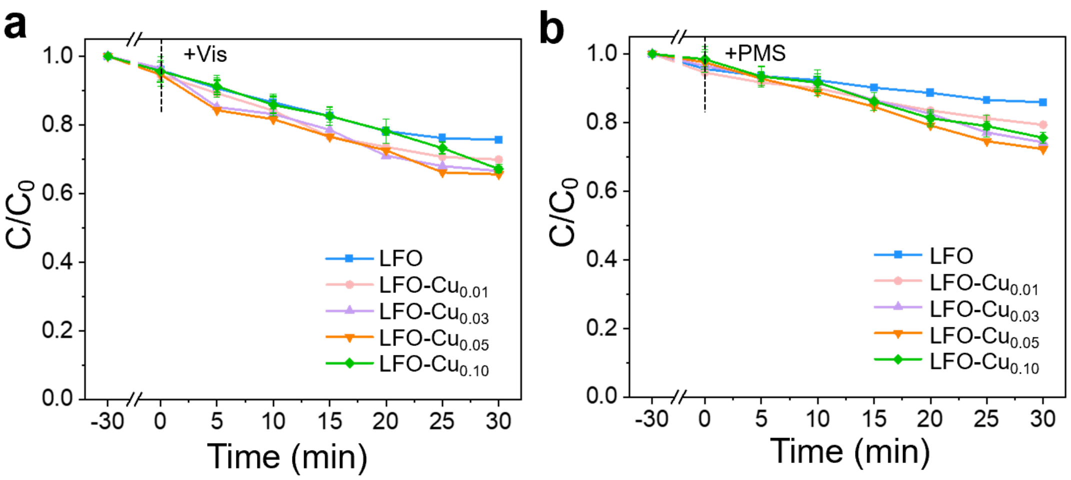

Supplementary Fig. 4 | Pre-experiments of different systems in beaker. Degradation of ONP with $LaFe_{1-x}Cu_xO_{3-\delta}$ (x = 0, 0.01, 0.03, 0.05, 0.10) in light irradiation (**a)** and PMS **(b)** system (λ > 420 nm, 50 mL 30 ppm ONP solution including 0.3 mM PMS and 0.4 g L^{-1} catalyst, initial pH = 7.1). Error bars are standard error values of three tests (n = 3).

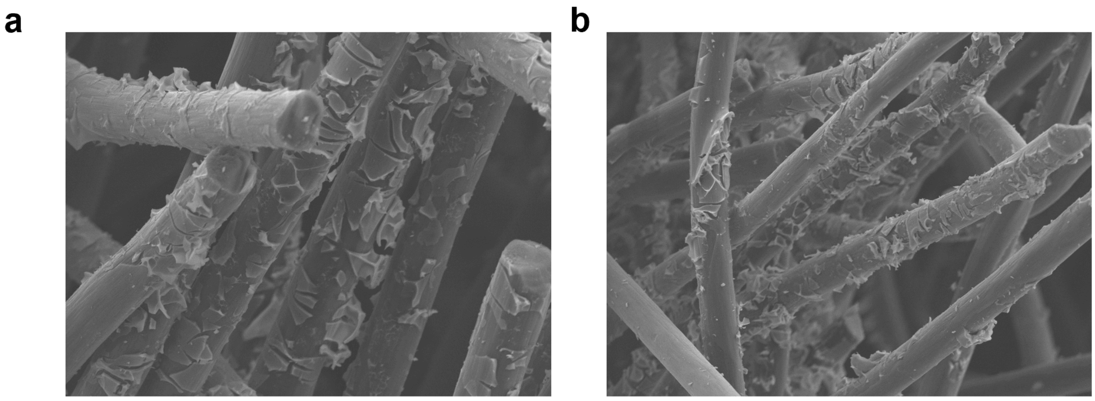

Supplementary Fig. 5 | Characterizations of LFCO@CFC. (a,b) SEM images of LFCO@CFC.

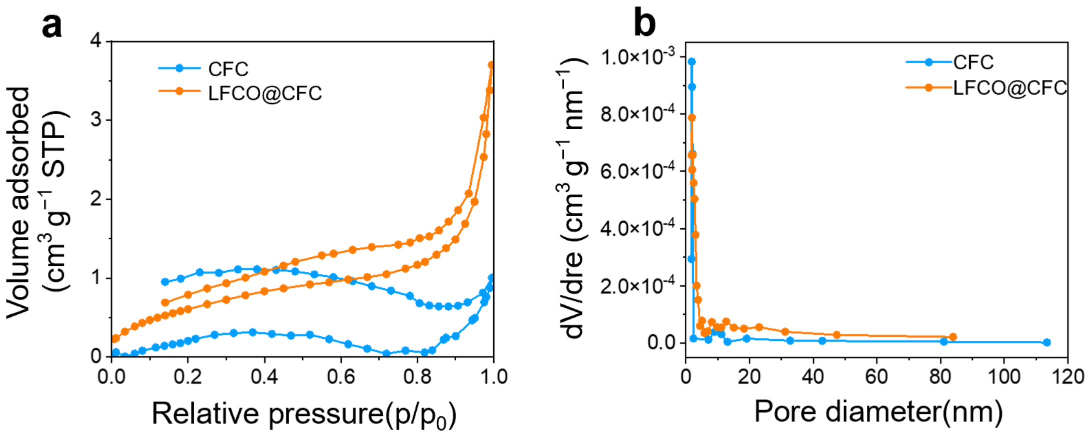

Supplementary Fig. 6 | BET characterization results of FBR. N_2 adsorption/desorption isotherms **(a)** and pore size distribution **(b)** of CFC and LFCO@CFC.

The N_2 adsorption-desorption isotherm of CFC and LFCO@CFC appertained to the typical type IV curve with type 3 hysteresis loop based on the IUPAC classification. The specific surface area of LFCO@CFC (2.2518 $m^2\ g^{-1}$) is slightly increased as compared with CFC (1.3685 $m^2\ g^{-1}$), which is beneficial for adsorption[13].

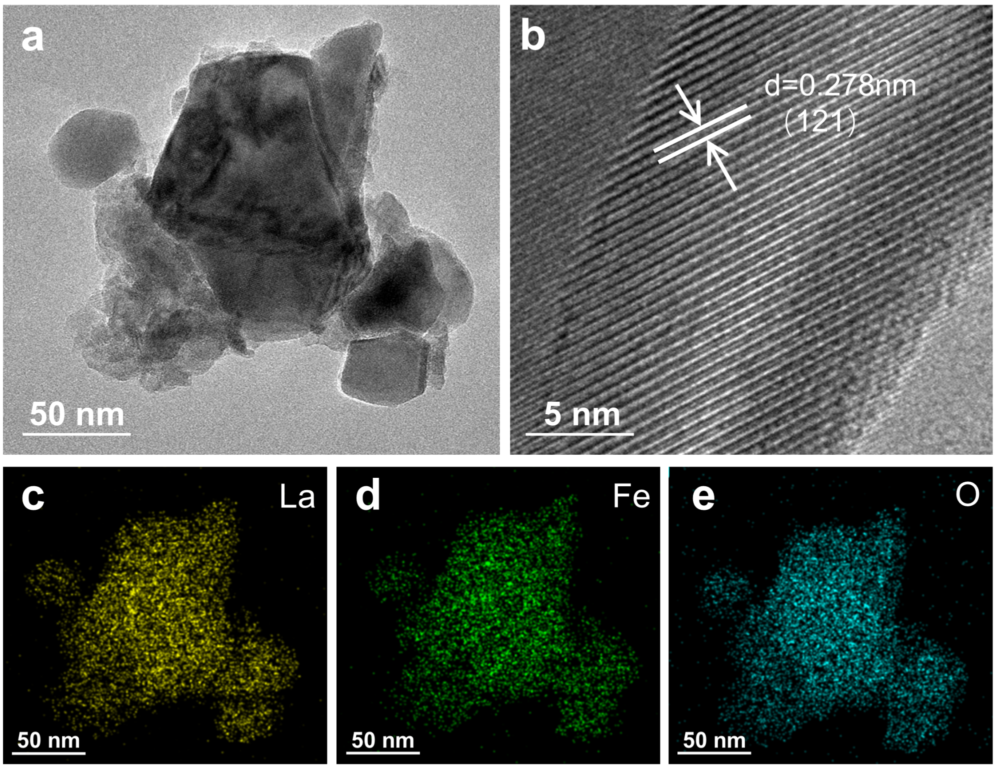

Supplementary Fig. 7 | Electron microscopic characterization of LFO. HRTEM images **(a)**, lattice fringes **(b),** and the EDS elemental mappings of La **(c)**, Fe **(d)**, and O **(c)** in LFO.

The main lattice striation spacing of 0.278 nm corresponds to the (121) crystal plane of cubic LFO, while the elemental mapping of each element also shows that La, Fe, and O are uniformly distributed in the crystal structure[14,15].

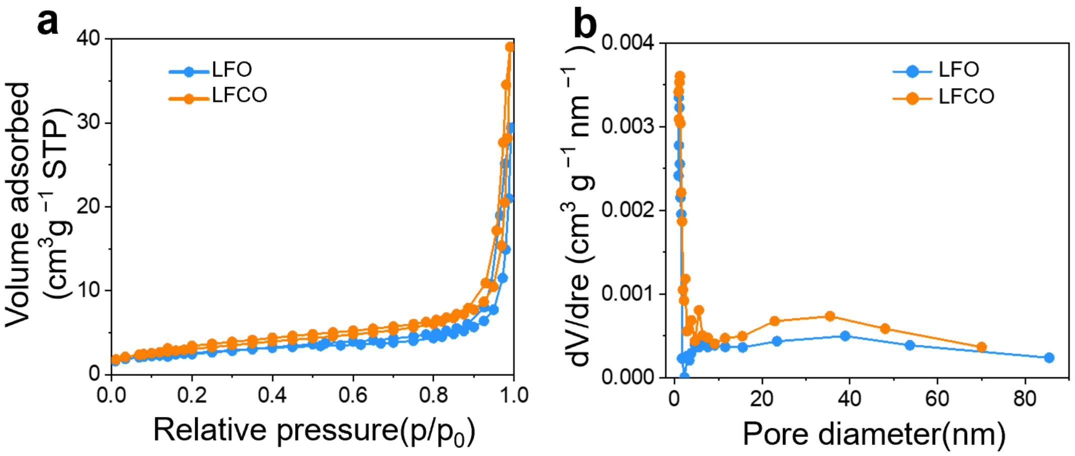

Supplementary Fig. 8 | BET characterization results of catalyst. N_2 adsorption/desorption isotherms (**a**) and pore size distribution (**b)** of LFO and LFCO.

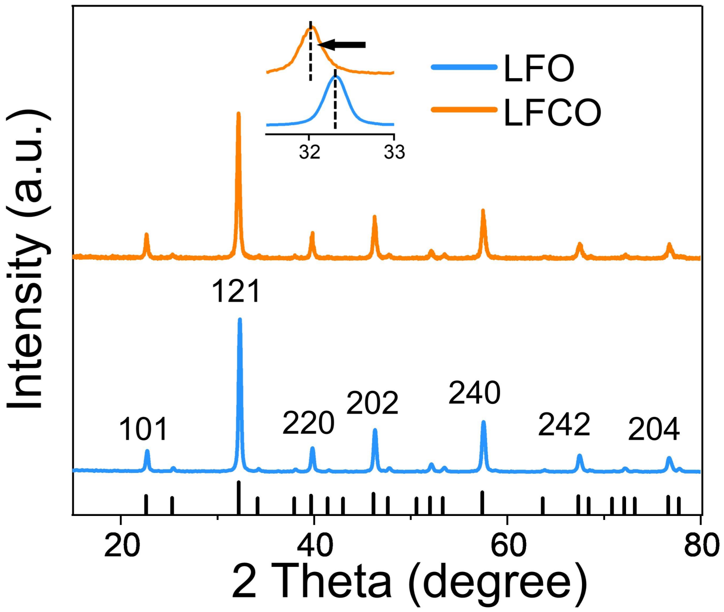

Supplementary Fig. 9 | XRD results of LFO and LFCO (inset figure: local amplification of the peak for LFO and LFCO at 121 plane)**.**

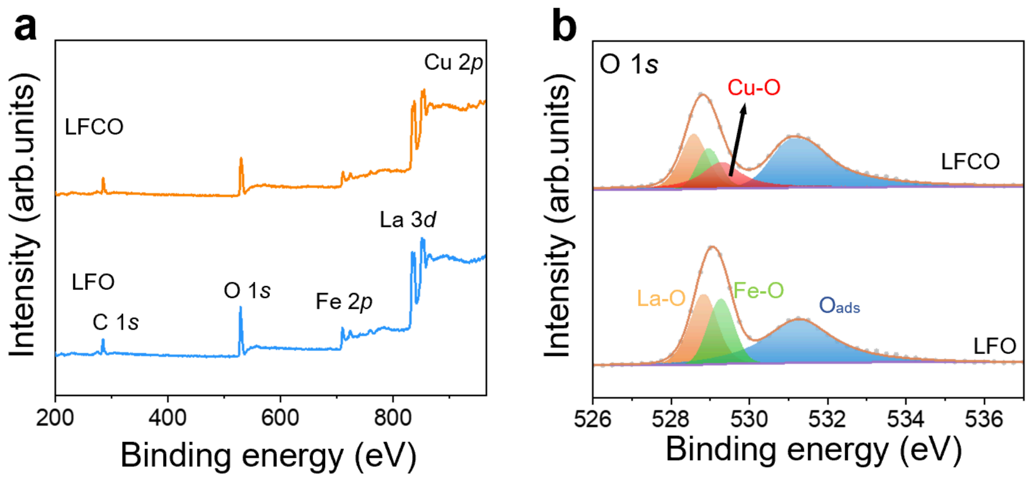

Supplementary Fig. 10 | XPS analysis of catalysts. The XPS survey spectra of LFO and LFCO **(a)**. The O 1*s* spectra of LFO and LFCO (The orange, green, red and blue shade represent the La-O, Fe-O, Cu-O, and adsorption oxygen (O_{ads}), respectively) **(b)**.

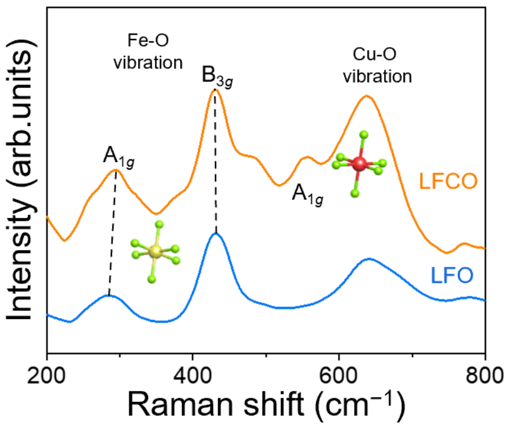

Supplementary Fig. 11 | Raman spectra of the LFO and LFCO. Peaks at 200-500 cm^{-1} belong to Fe-O vibration (A_{1g} and B_{3g}) and 600-700 cm^{-1} belong to Cu-O vibration (A_{1g}).

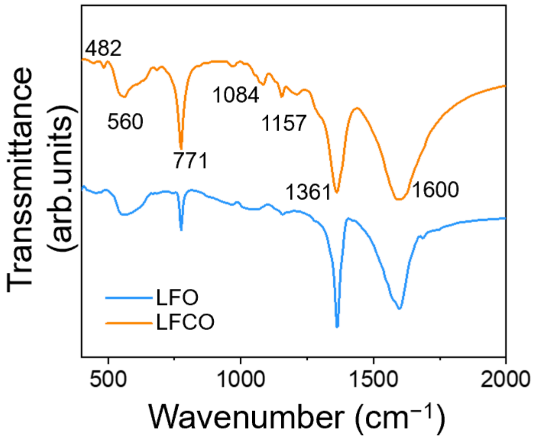

Supplementary Fig. 12 | FTIR analysis of catalysts. FTIR spectra of LFO and LFCO.

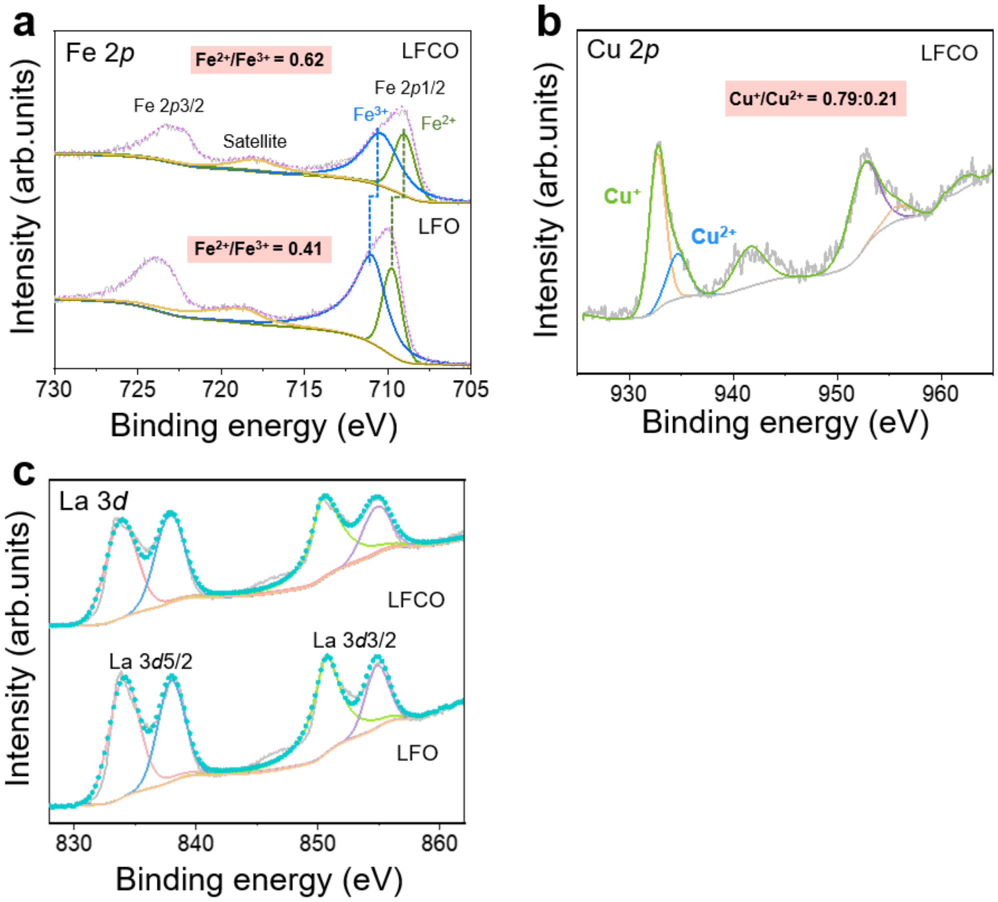

Supplementary Fig. 13 | XPS analysis of catalysts. The XPS La 3*d* **(a)**, Fe 2*p* **(b)** and Cu 2*p* **(c)** spectra of LFO and LFCO.

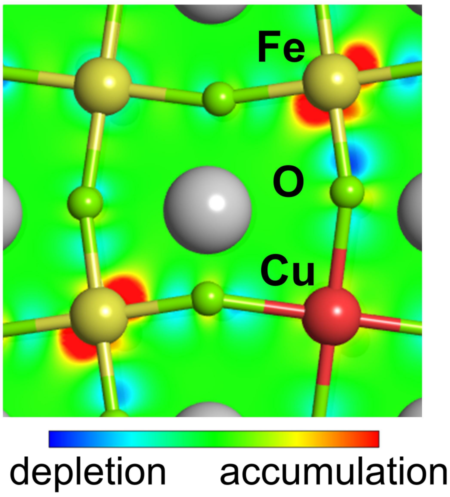

Supplementary Fig. 14 | Charge distribution analysis of catalysts. The charge distribution in LFCO.

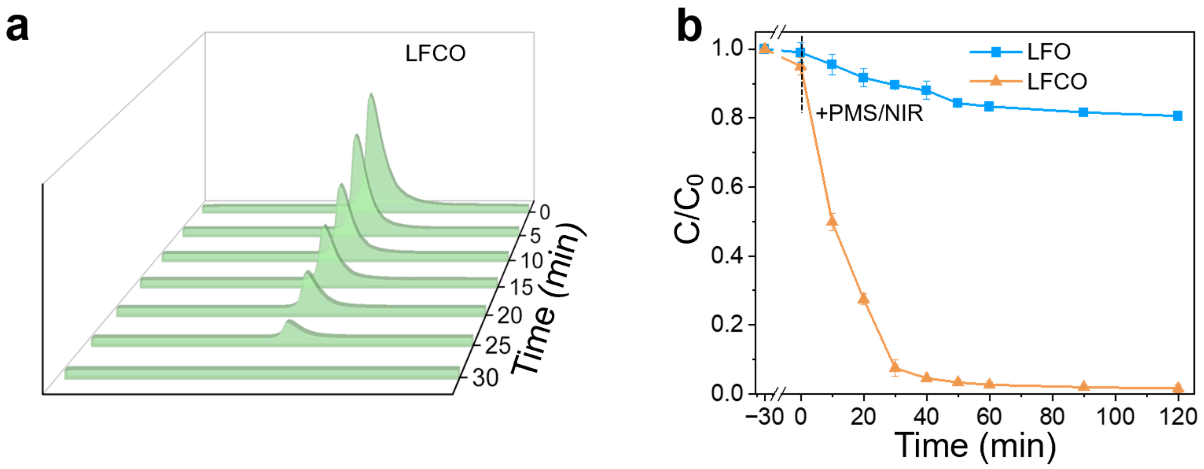

Supplementary Fig. 15 | Degradation performance in PMS/NIR system. The HPLC results for the degradation of ONP in the LFCO/PMS/NIR system **(a)**. The calculated ONP degradation efficiency with adding LFCO and LFO **(b)**. Conditions: λ > 780 nm, 50 mL 30 ppm ONP solution, 0.3 mM PMS, 0.3 g L^{-1} powder catalyst, 298 K, initial pH 7.1. Error bars are standard error values of three tests (n = 3).

Based on the results from HPLC, a higher degradation rate (95%) of ONP was achieved in 30 min by applying LFCO, while only 10% of ONP was degraded for LFO.

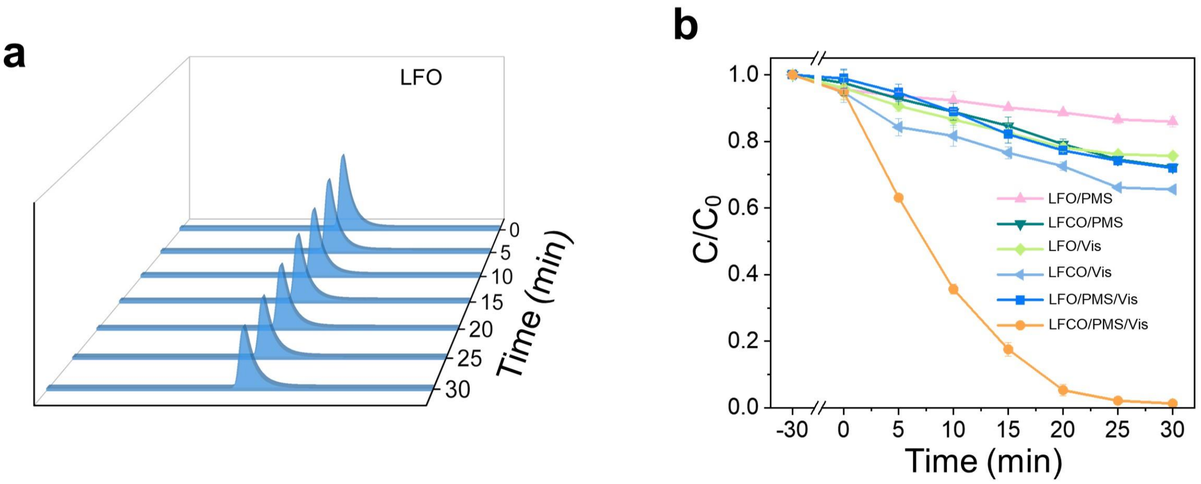

Supplementary Fig. 16 | Degradation performance in PMS/Vis system. The HPLC profiles for the degradation of ONP in LFO/PMS/Vis system **(a)**. The degradation rate of ONP of different systems **(b)**. Conditions: $\lambda > 420$ nm, 50 mL 30 ppm ONP solution, 0.3 mM PMS, 0.3 $g \cdot L^{-1}$ powder catalyst, 298 K, initial pH 7.1. Error bars are standard error values of three tests (n = 3).

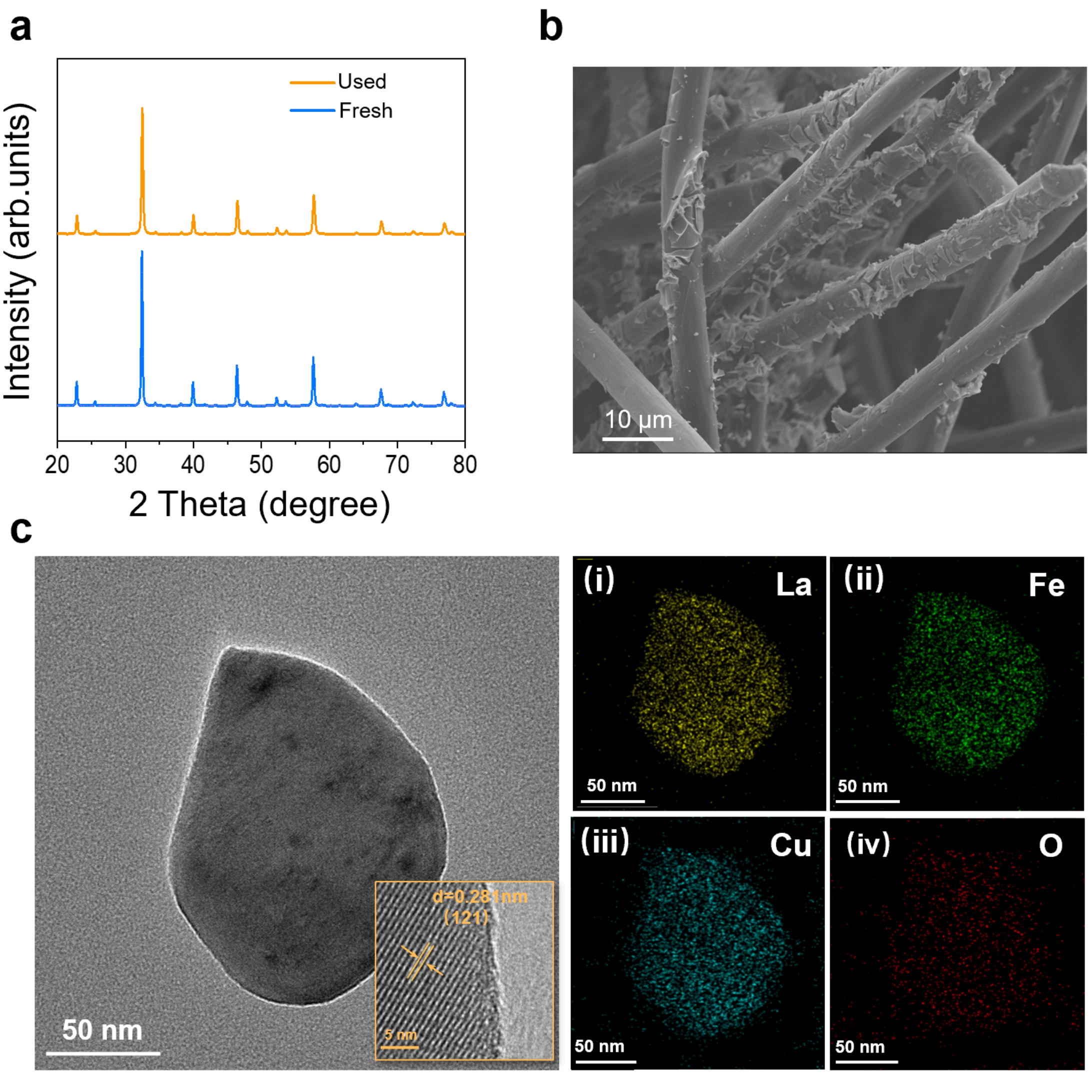

Supplementary Fig. 17 | Characterizations of used LFCO. XRD **(a)**, SEM **(b)**, TEM images (inset: TEM in 5 nm with lattice fringe) **(c)** and corresponding EDS spectra **(i-iv)** of used LFCO.

The intensity/half peak width of XRD peaks is not only dependent on the particle size but also the content of the crystal phase. After a 7-day catalytic reaction, we can see that the used LFCO catalyst shows the same peaks as fresh LFCO (Supplementary Fig. 17a), indicating that no phase transition during the reaction. As shown in SEM results (Supplementary Fig. 17b), the catalyst is still tightly bound to CFC, indicating the active components were not lost with time. Besides, TEM results of used LFCO catalysts show that they maintained the particle structure and size with a clean surface and uniform distribution of elements, which is well agreed with XRD data. These results together proved the structure stability of LFCO catalysts.

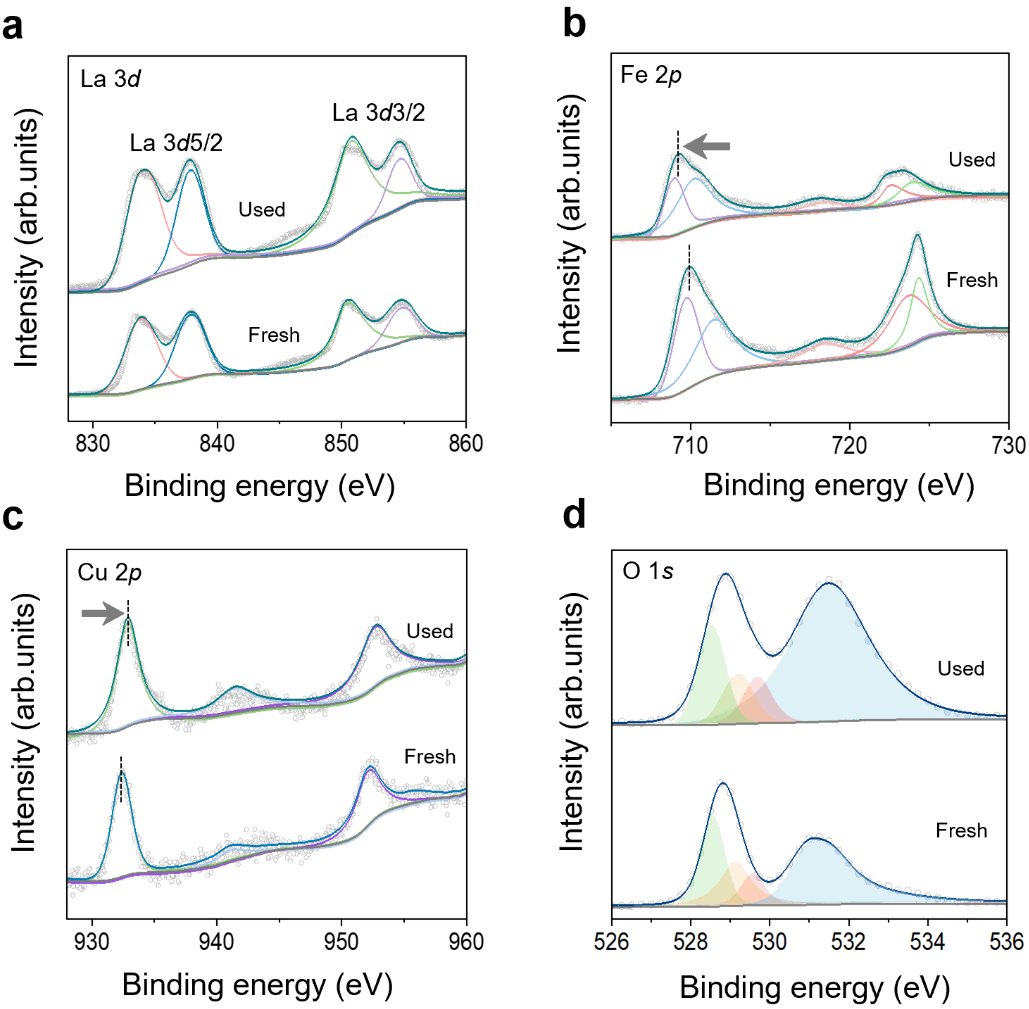

Supplementary Fig. 18 | XPS tracking of catalyst before and after reaction. The XPS La 3*d* **(a)**, Fe 2*p* **(b)**, Cu 2*p* **(c)**, and O 2*p* **(d)** spectra of fresh and used LFCO. The green, orange, red and blue shade in Supplementary Fig. 18d represent the La-O, Fe-O, Cu-O, and adsorption oxygen (O_{ads}), respectively.

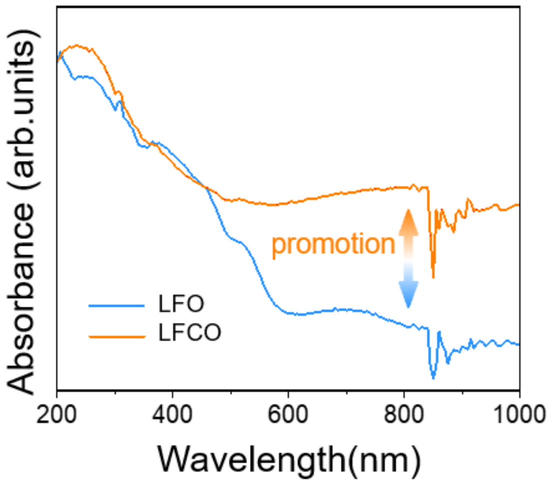

Supplementary Fig. 19 | UV-Vis DRS analysis of catalysts. UV-Vis DRS spectra of LFO and LFCO.

We found that LFCO has a relatively obvious response in the near-infrared wavelength range, proving the higher degradation rate (95%) of ONP using LFCO compared with LFO (10%) in Supplementary Fig. 15.

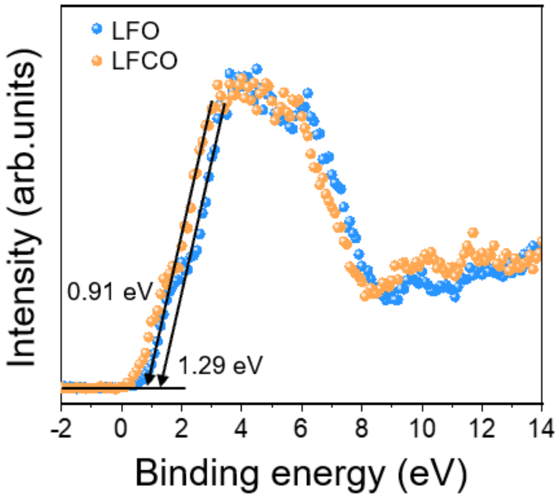

Supplementary Fig. 20 | Valence band analysis of materials. The valence band of LFO and LFCO.

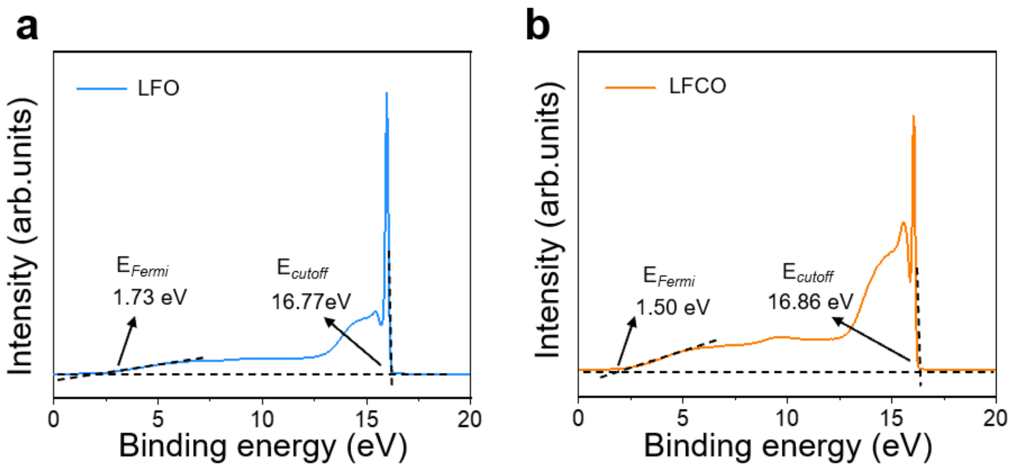

Supplementary Fig. 21 | UPS analysis of materials. UPS spectra for LFO (a) and LFCO (b).

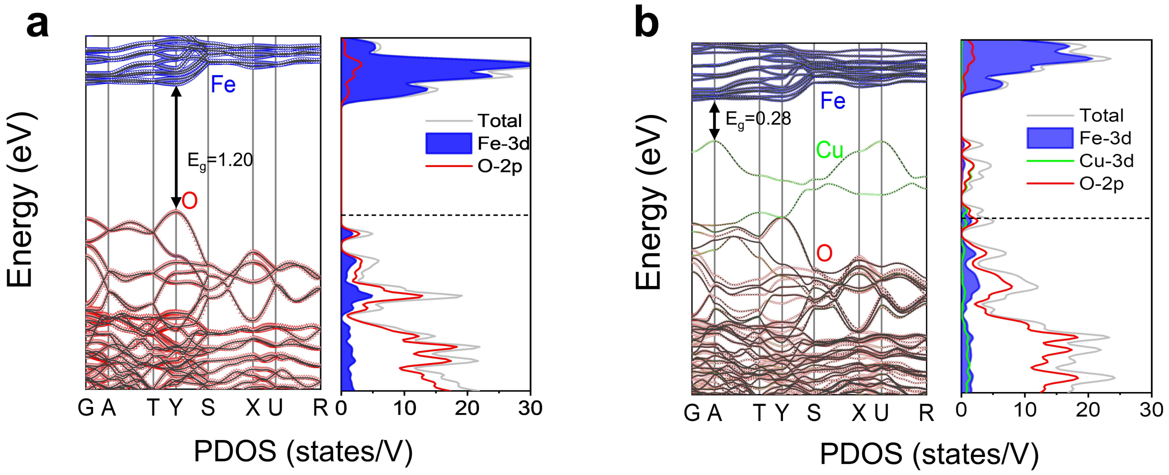

Supplementary Fig. 22 | Band structure analysis of materials. The calculated DOS and projected band structure of LFO (a) and LFCO (b).

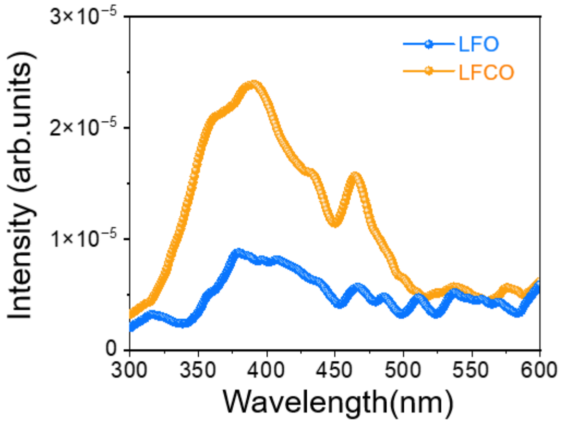

Supplementary Fig. 23 | Surface electronic structure analysis. SPV responses of LFO and LFCO.

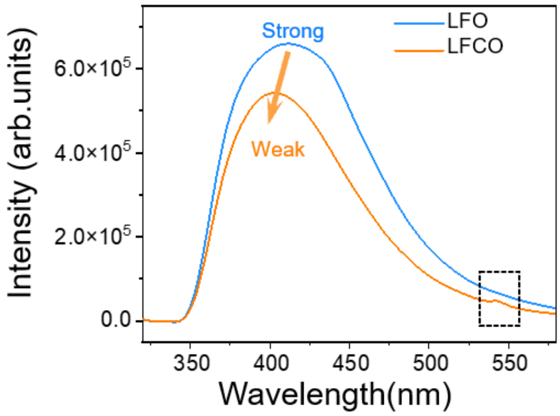

Supplementary Fig. 24 | Surface electronic structure analysis. Steady-state PL responses of LFO and LFCO.

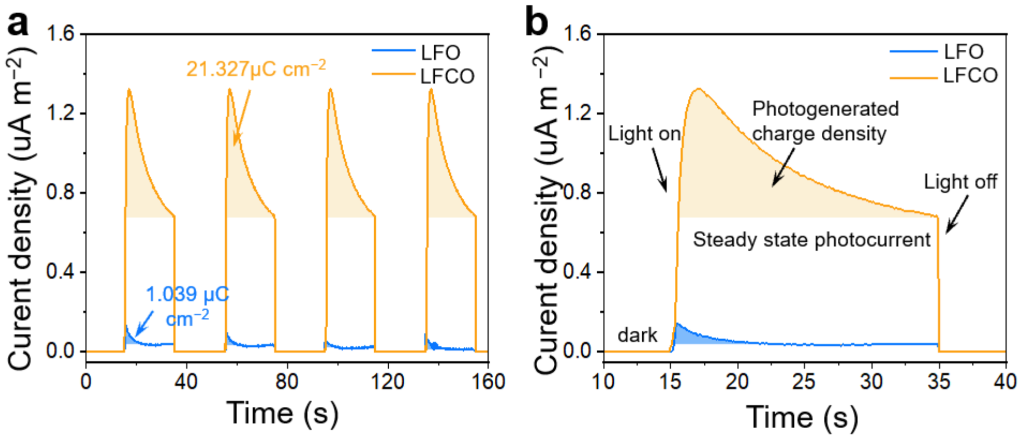

Supplementary Fig. 25 | TPC (a) and the corresponding surface charge density (b) of LFO and LFCO.

(The orange shade represents the photogenerated charge density, method for calculation was listed in Supplementary Methods)

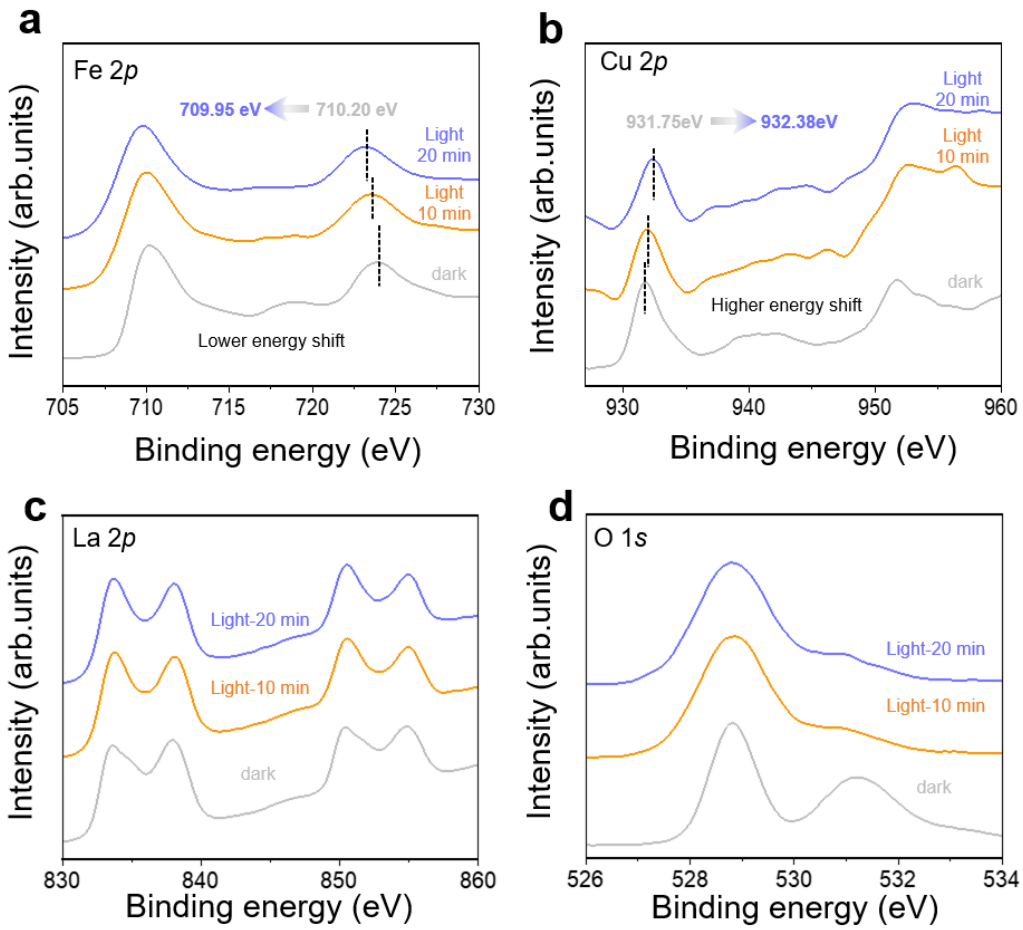

Supplementary Fig. 26 | XPS tracking of catalyst during reaction. In situ XPS of Fe 2*p* **(a)**, Cu 2*p* **(b)**, La 3*d* **(c)**, O 1*s* **(d)** spectra of LFCO.

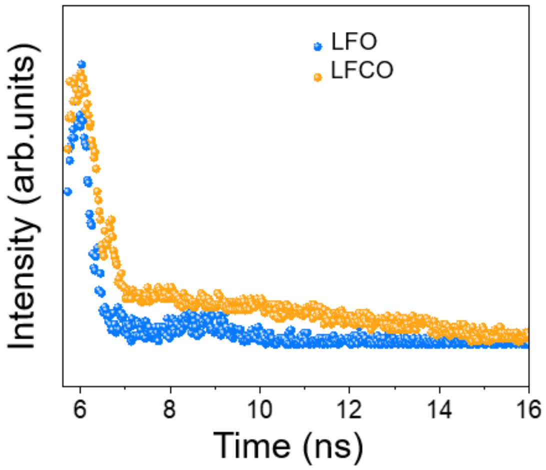

Supplementary Fig. 27 | Spectral analysis of materials. TRPL spectra of LFO and LFCO.

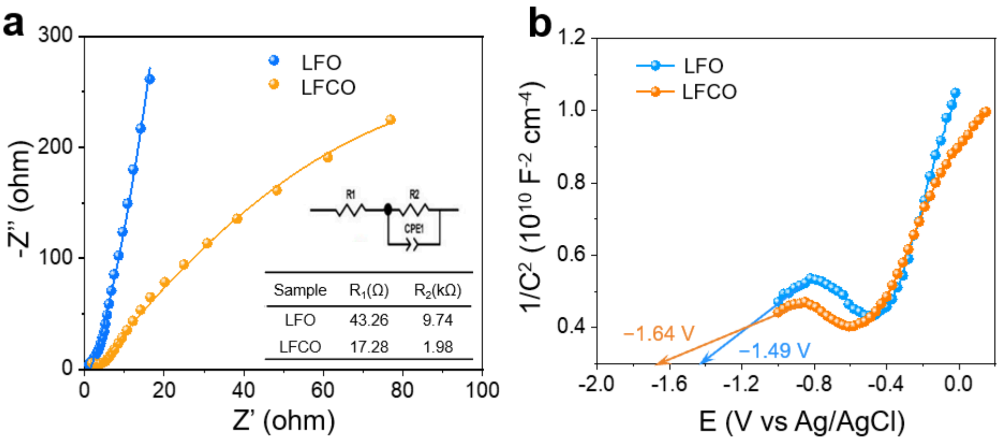

Supplementary Fig. 28 | Electrochemical analysis of materials. EIS (inset: analog circuit diagram) (a) and M-S plots (b) of LFO and LFCO.

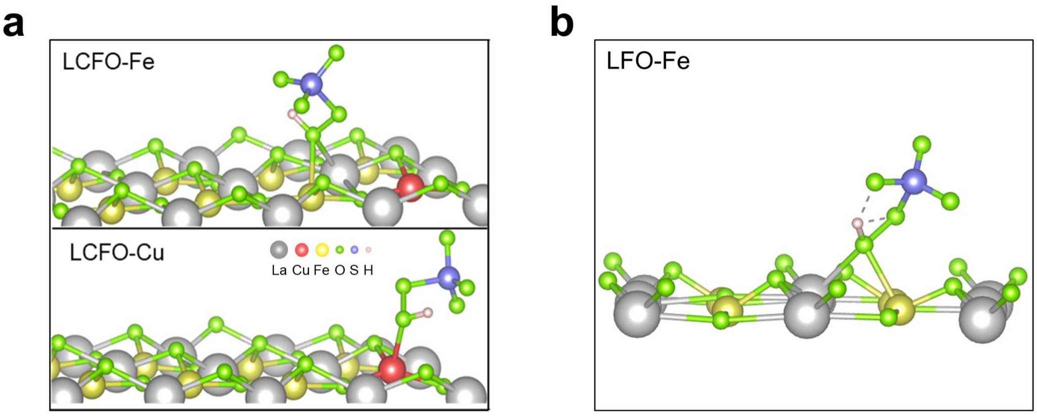

Supplementary Fig. 29 | Molecular adsorption structure analysis. The optimized adsorption configurations of PMS molecules on the surface of the LFCO (a) and LFO (b) model.

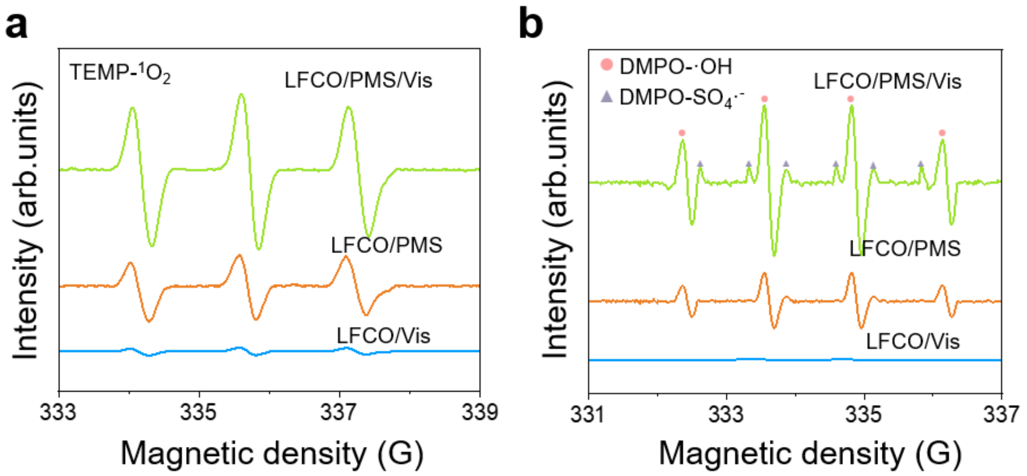

Supplementary Fig. 30 | Spin-trapping ESR spectra for detecting $^1O_2$ and •OH/$SO_4^{•-}$. ESR spectra of TEMP-$^1O_2$ (**a**) and DMPO-•OH/$SO_4^{•-}$ (**b**) in LFCO/Vis, LFCO/PMS, and LFCO/PMS/Vis system.

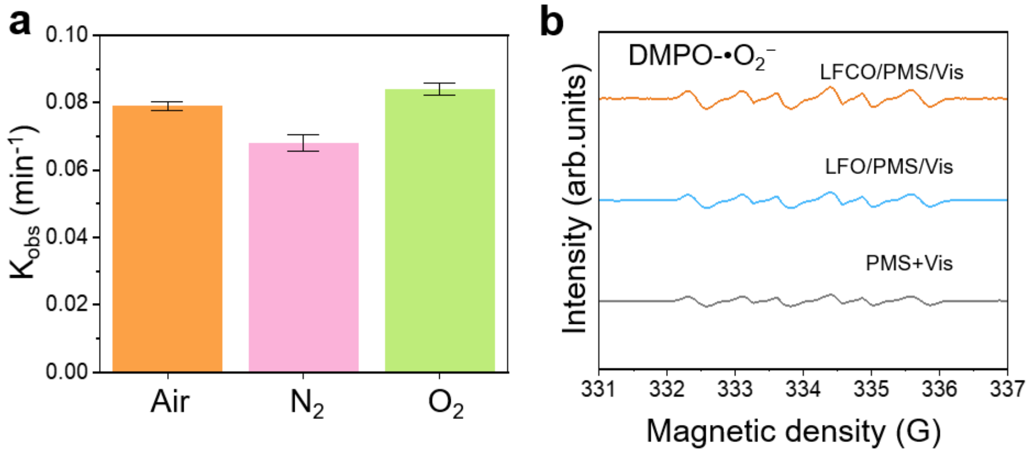

Supplementary Fig. 31 | Experiments and ESR spectra for detecting •O_2^-. The degradation of ONP of LFCO/PMS/Vis system in the different atmospheres (Air, N_2, O_2) **(a)**. Error bars are standard error values of three tests (n = 3). Spin-trapping ESR spectra of DMPO-•O_2^- in different catalysts systems **(b)**.

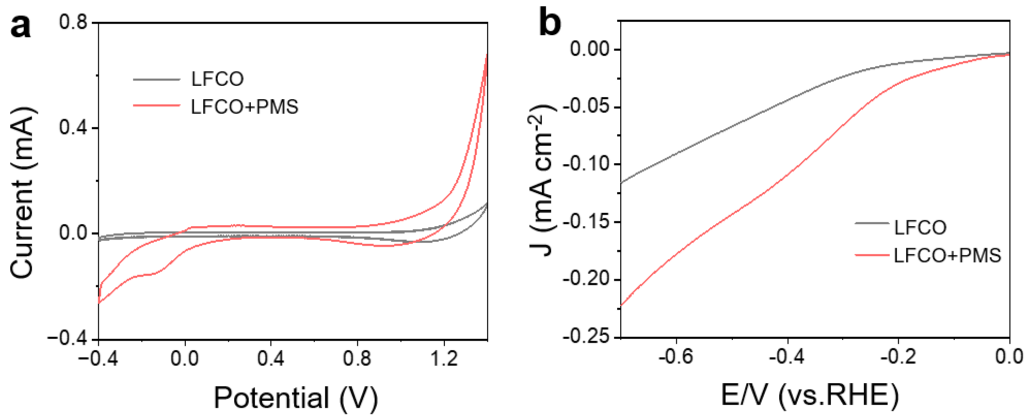

Supplementary Fig. 32 | Electrochemical analysis of materials. CV (a) and LSV (b) measurement of LFCO with/without adding PMS.

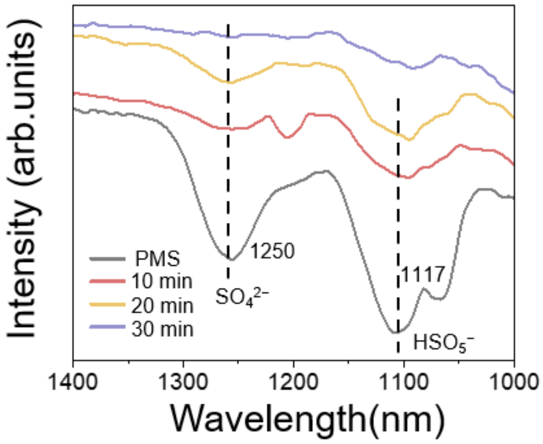

Supplementary Fig. 33 | Analysis of catalytic reaction process. In situ ATR-FTIR spectra of LFCO in PMS/Vis system during reaction. Two bands appeared at 1117 and 1250 cm^{-1} belonging to S-O tensile vibration of SO_4^{2-} and HSO_5^-, respectively.

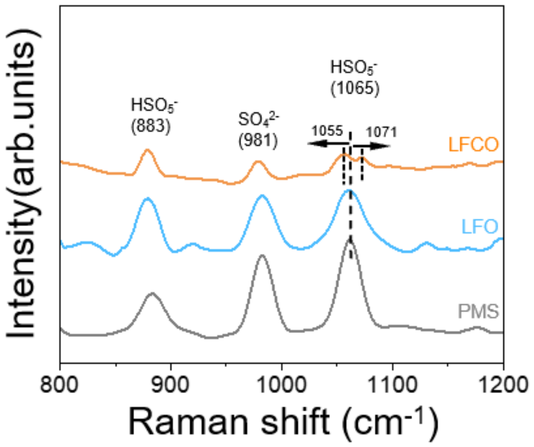

Supplementary Fig. 34 | Analysis of catalytic reaction process. In situ Raman spectra of LFCO in PMS/Vis system during reaction. The peaks at 883, 981, and 1065 cm^{-1} represent the stretching vibrations of O-O bonds in HSO_5^-, S=O bonds in SO_4^{2-}, and SO_3 in HSO_5^-.

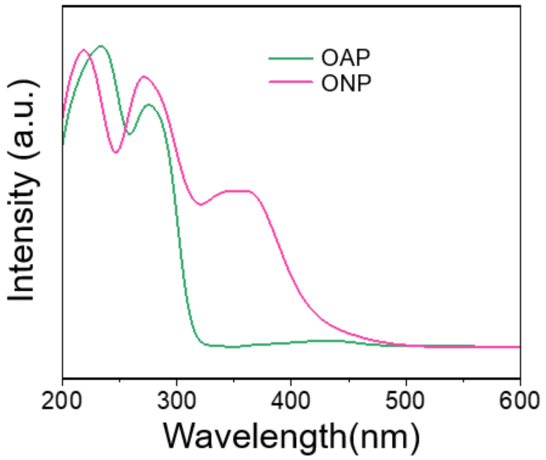

Supplementary Fig. 35 | Spectral analysis of materials. UV-vis absorption spectra of ONP and OAP.

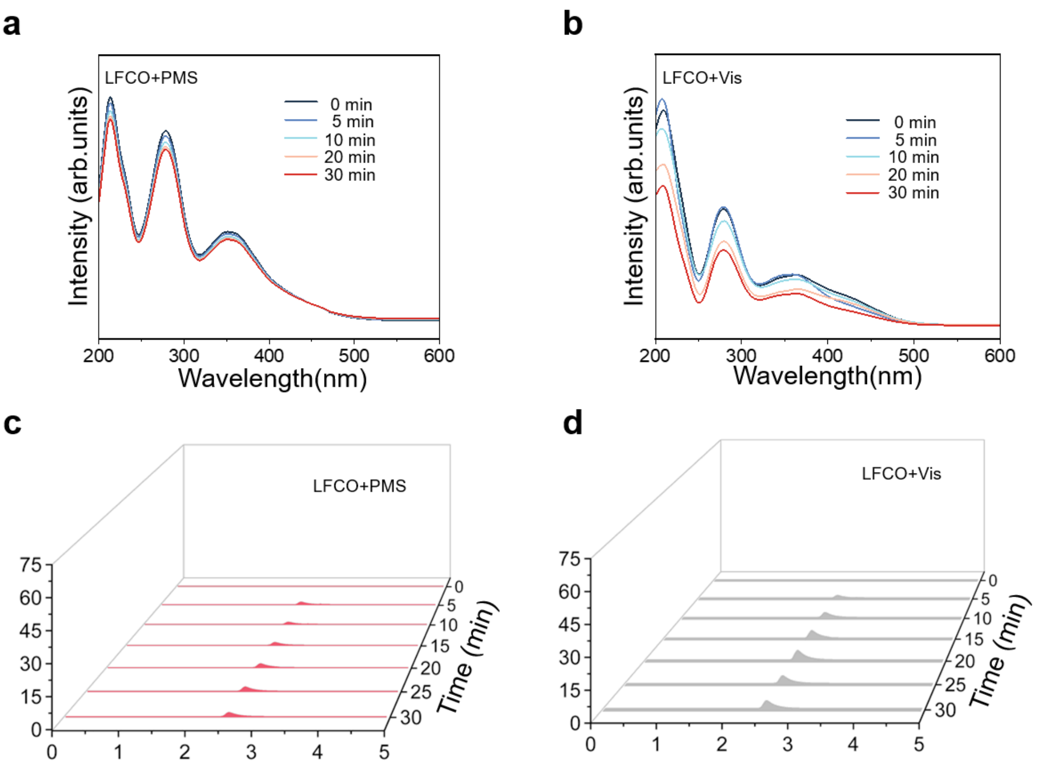

Supplementary Fig. 36 | Identification of reduction products of ONP during reaction. In situ UV-vis absorption spectra of ONP degradation in the LFCO/PMS **(a)** and LFCO/Vis **(b)** system. HPLC spectra of generated OAP in the LFCO/PMS **(c)** and LFCO/Vis **(d)** system.

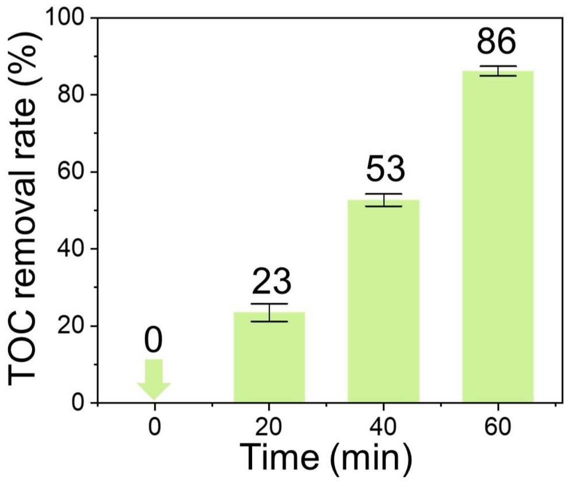

Supplementary Fig. 37 | The TOC removal rate in the LFCO/PMS/Vis system during ONP degradation.

Error bars are standard error values of three tests (n = 3).

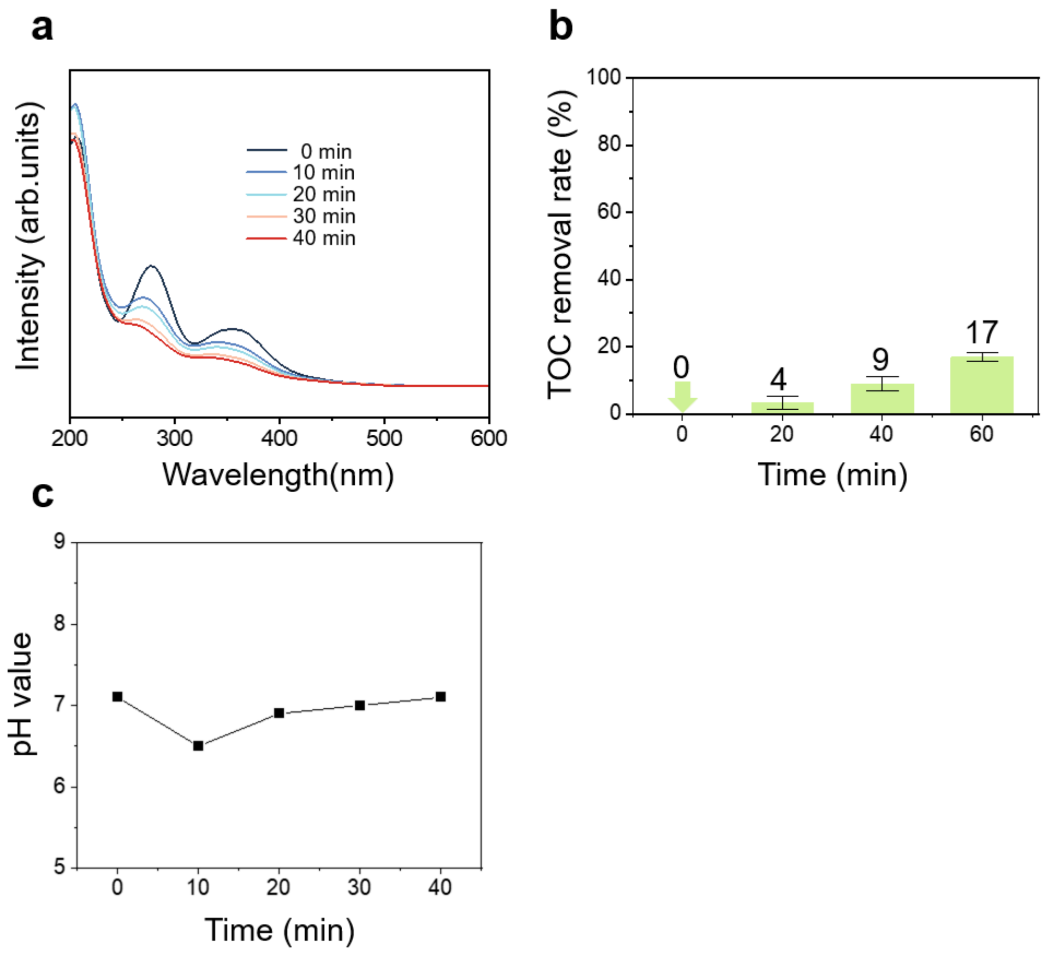

Supplementary Fig. 38 | Identification of reduction products of ONP in the LFCO/PMS/NIR system. UV-Vis absorption spectra **(a)**, the TOC removal rate **(b)**, and the changes of pH (**c**) during the ONP degradation process in the LFCO/PMS/NIR system. Error bars are standard error values of three tests (n = 3).

Clearly shown in Supplementary Fig. 38c, the pH of the reaction solution changes from neutral to weakly acidic during the reduction stage (0 ~ 10 min), which is conducive to providing H^+. It returns to neutral as the reaction proceeds. One of the reasons for the phenomenon may be that the PMS oxidization process could contribute to the H^+ according to Eq. (3) in the manuscript.

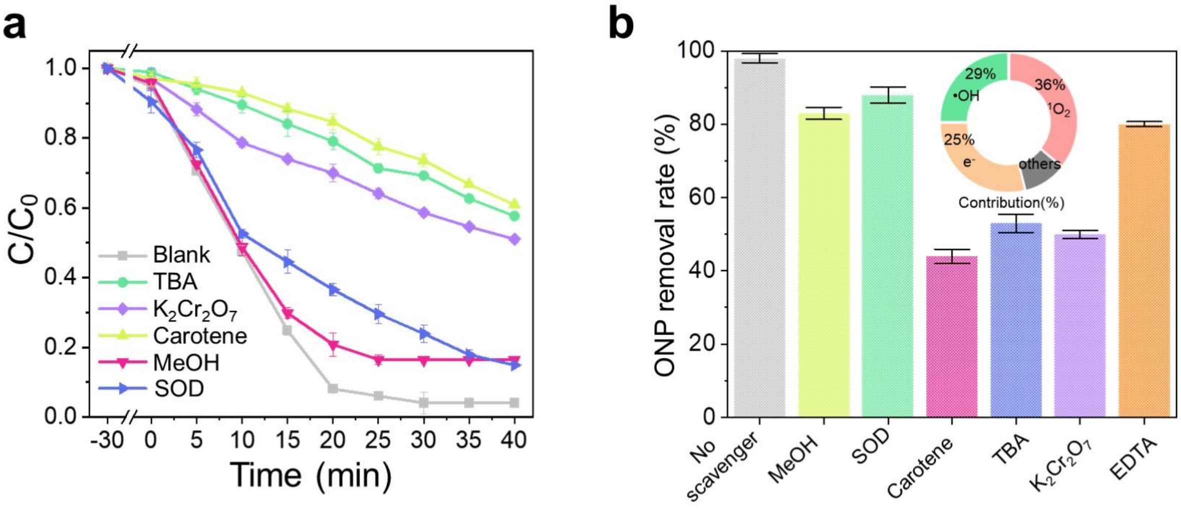

Supplementary Fig. 39 | Quenching experiments. The ONP degradation performance at different time intervals (**a)** and corresponding ONP removal rate (inset figure: contributions of different ROS) **(b)** with adding different scavengers in LFCO/PMS/Vis system. Conditions: $\lambda > 420$ nm, 50 mL 30 ppm ONP solution, 0.3 mM PMS, 0.3 $g \cdot L^{-1}$ powder catalyst, 298 K, initial pH 7.1. 0.05 mmol L^{-1} scavengers were added to the solution before light irradiation. Error bars are standard error values of three tests ($n = 3$).

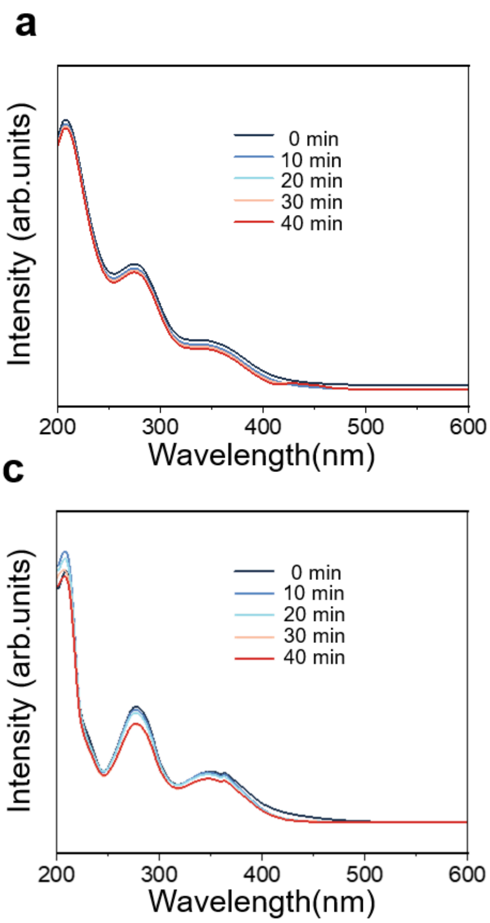

Supplementary Fig. 40 | Role of reactive species in ONP degradation. Typical UV-vis absorption spectra of the affection of scavengers of e^- **(a)**, $^1O_2$ **(b)**, and •OH **(c)** during ONP degradation in LFCO/PMS/Vis system.

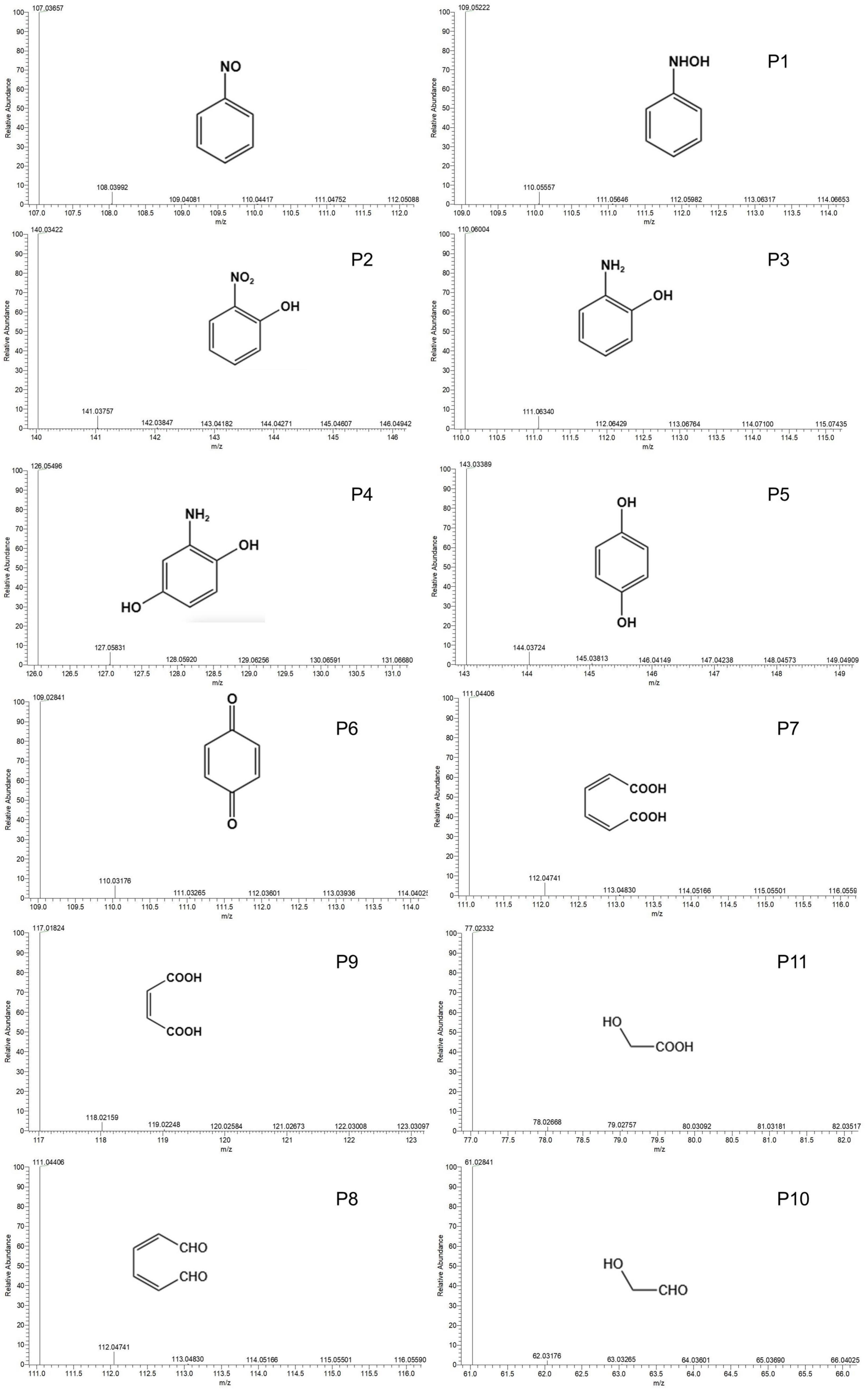

Supplementary Fig. 41 | Structure analysis of intermediates. Mass spectra and the corresponding chemical structure of ONP and intermediates (P1-P11) during the ONP degradation.

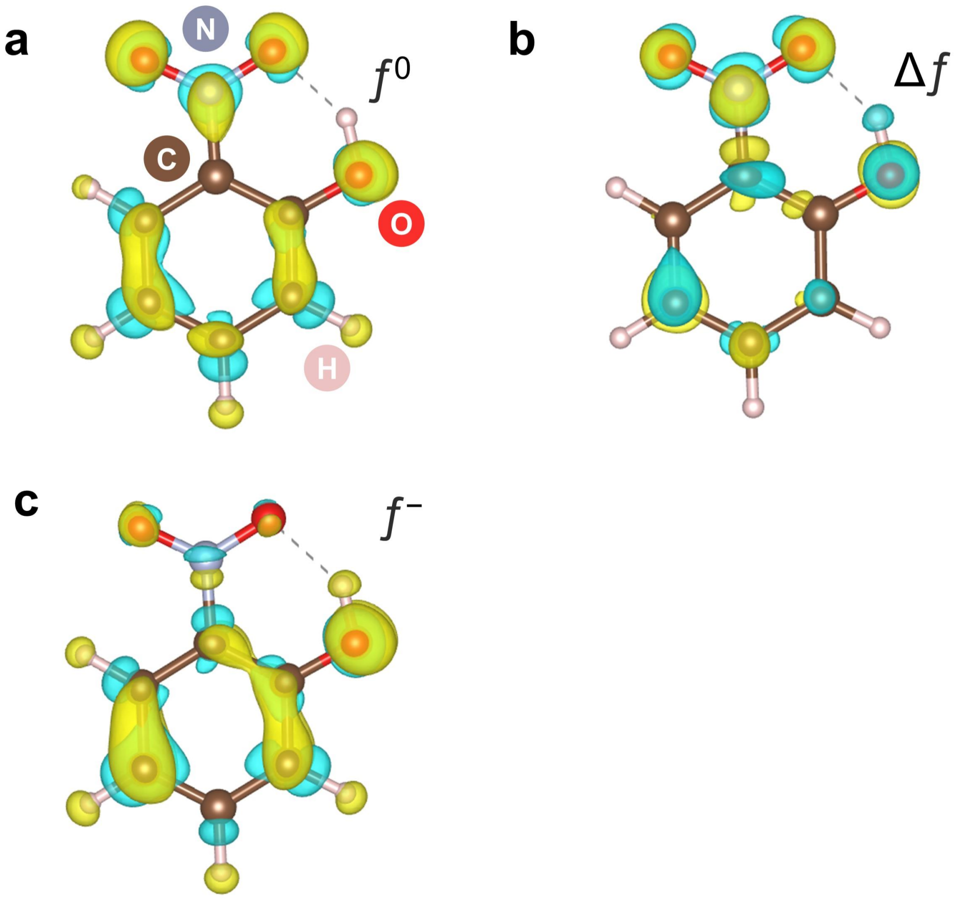

Supplementary Fig. 42 | Fukui index distributions. f^0 **(a)**, CDD **(b)** and f^- **(c)** of ONP. Yellow and cyan regions represent the electron accumulation and the electron depletion, respectively.

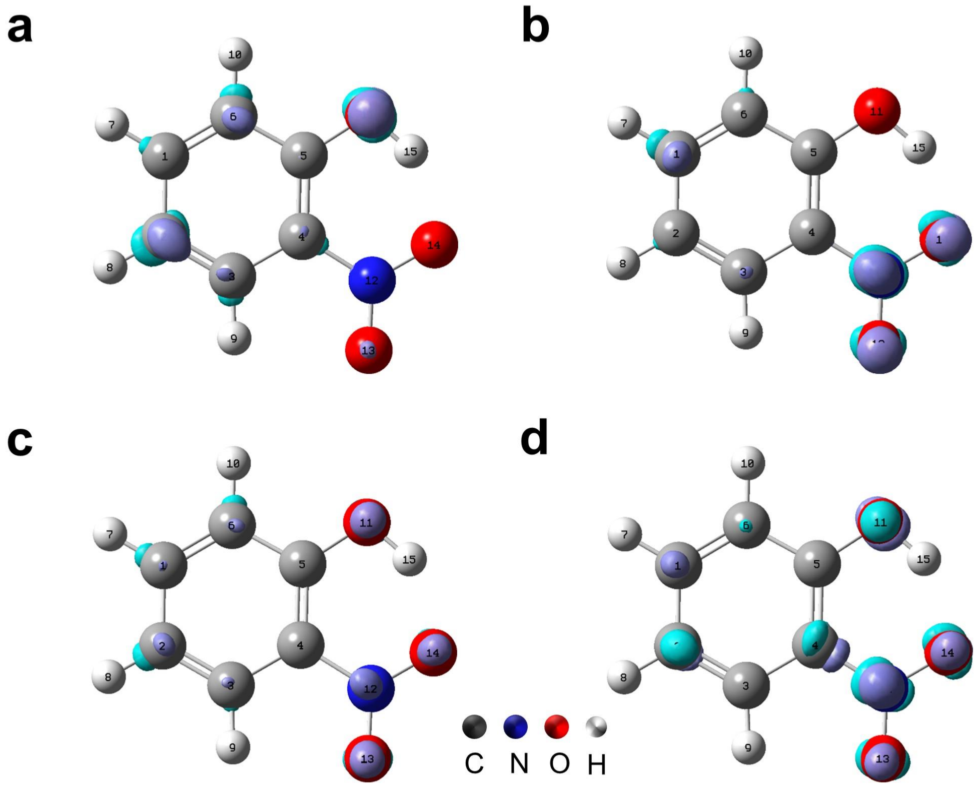

Supplementary Fig. 43 | Fukui function of ONP molecule. **a** f^+ **b** f^- **c** f^0 **d** Δf at isosurface level equal to 0.01 a.u. Purple and green correspond to positive and negative places in the orbit calculation, respectively.

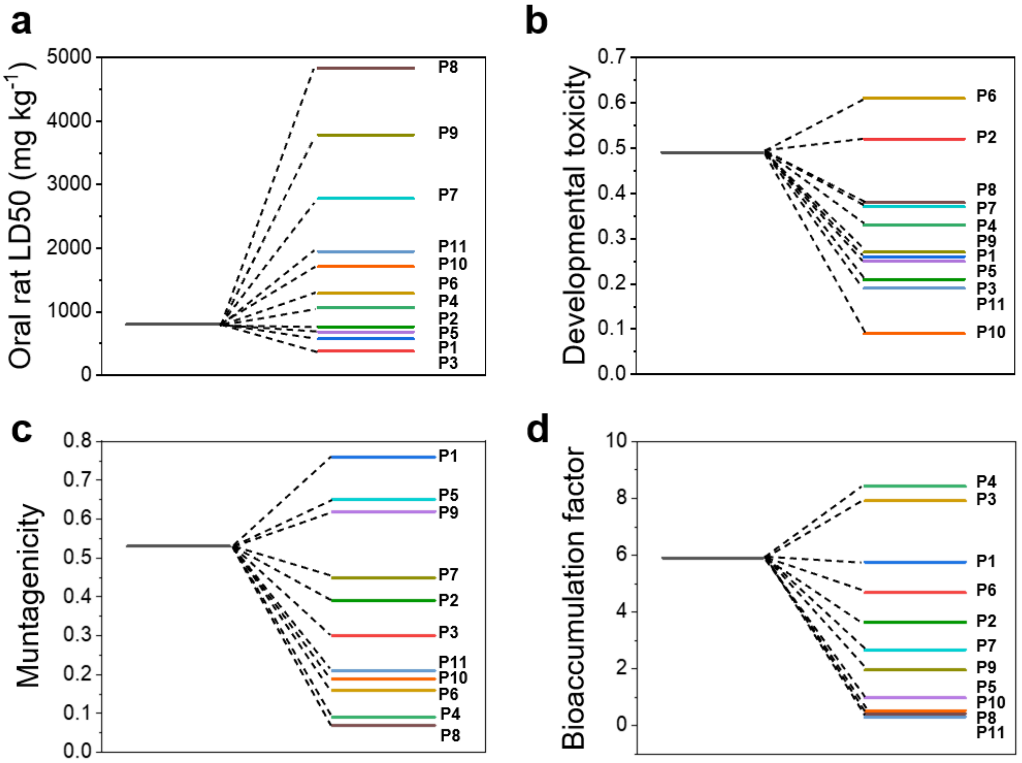

Supplementary Fig. 44 | Toxicity assessment. Oral rat LD50 **(a)**, Developmental **(b)**, Toxicity mutagenicity **(c)** and Bioaccumulation factor **(d)** of parent ONP and its degradation products. All the chemical structures were shown in Supplementary Fig. 41.

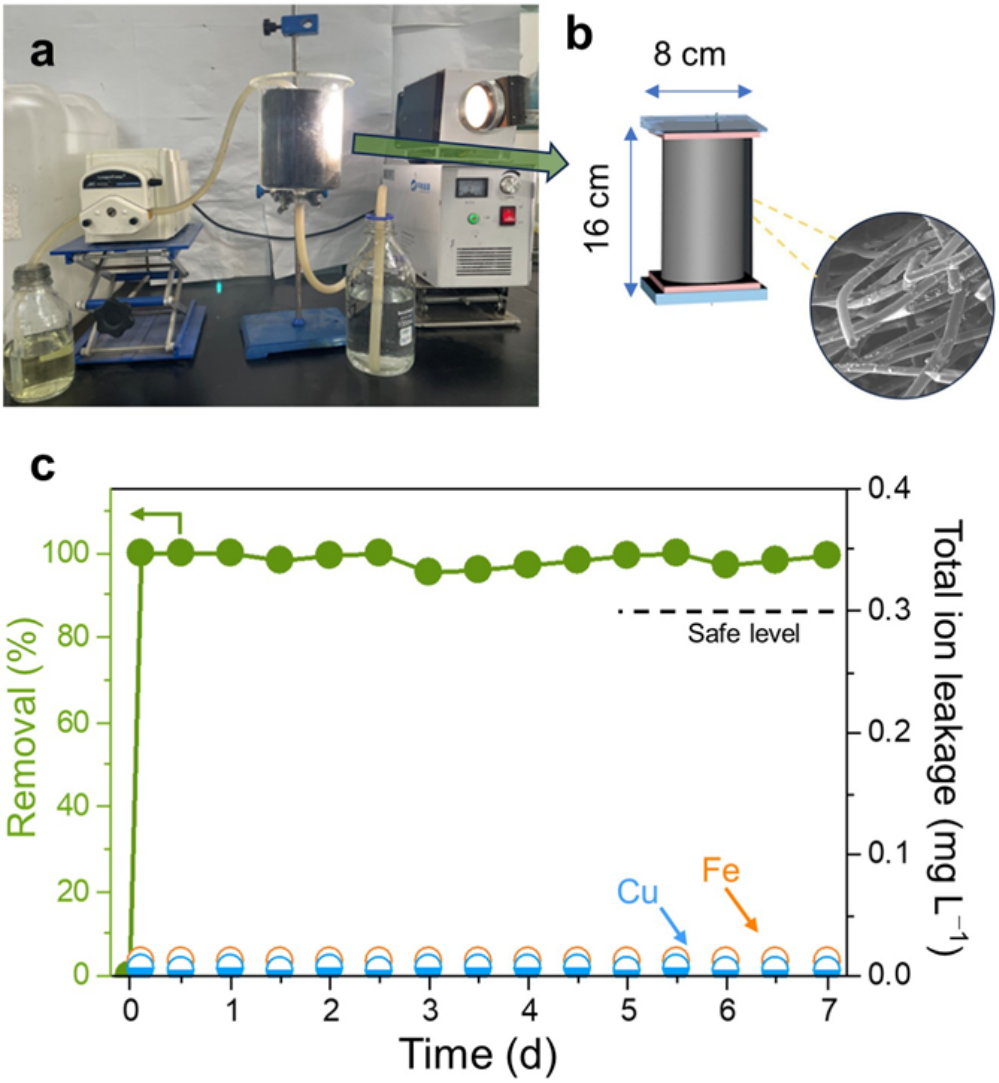

Supplementary Fig. 45 | Scaled-up application. Scaled-up setup of the LFCO@CFC FBR systems with the fixed bed reactor (diameter: 8 cm, high: 16 cm), Xe lamp, and peristaltic pump **(a)**. Structure diagram of the fixed bed reactor **(b)**. Long-term operation performance **(c)**. Conditions (unless indicated otherwise): $\lambda > 420$ nm radiation, Water flux 1500 mL h^{-1}, HRT 60 s, 298 K, initial pH 7.1, 30 ppm ONP, and 3 mM PMS (introduced with the ONP stock solution).

3. Supplementary Tables

Supplementary Table 1 | BET surface area and pore volume of LFO and LFCO.

Catalyst	BET surface area (m^2/g)
LFO	8.09
LFCO	10.69

Supplementary Table 2 | Cu loading of different catalysts detected by ICP-MS

Catalyst	Cu atomic ratio (%)
LFO	0
LFCO	4.43

Supplementary Table 3 | The refined lattice parameters and reliability factors of LFO, LFCO samples.

Catalyst	Phase	Space group	Lattice parameters				R_p	R_{wp}
			a (Å)	b (Å)	c (Å)	V (Å^3)	(%)	(%)
LFO	orthorhombic	*Pnma*	5.570	7.866	5.563	243.74	5.68	9.71
LFCO			5.574	7.868	5.564	244.04	6.25	9.78

a, b, and c are the length of the unit translation vector in the direction of the three crystal axes, respectively.

R_p, R-pattern, pattern variance factor;

R_{wp}, R-weighted pattern, and weighted graph variance factor, both of which describe fitted graphs.

We can find a stable c/a ratio for LFO (0.9986) and LFO (0.9982), representing the unchanged phase type. Also, the R_p of LFO and LFO is 5.68% and 6.25%, respectively, which shows that the fitting results are reliable.

Supplementary Table 4 | Elements ratio of catalyst detected by XPS.

Catalyst	La (atomic %)	Cu (atomic %)	Fe (atomic %)	O (atomic %)
LFO	13.30	0	22.91	63.79
LFCO	14.76	4.61	17.36	63.27
LFCO-used	14.47	4.59	17.41	63.53

Supplementary Table 5 | Fukui function of ONP molecule at iso-surface level equal to 0.01 a.u.

Atom	q(N)	q(N+1)	q(N-1)	f^-	f^+	f^0	CDD
1(C)	-0.0146	-0.1051	0.0362	0.0508	0.0904	0.0706	0.0396
2(C)	-0.0462	-0.0949	0.0874	0.1336	0.0488	0.0912	-0.0848
3(C)	-0.0285	-0.0893	0.0485	0.0771	0.0607	0.0689	-0.0163
4(C)	0.0003	-0.0236	0.0580	0.0576	0.0240	0.0408	-0.0337
5(C)	0.0994	0.0537	0.1754	0.0760	0.0457	0.0608	-0.0303
6(C)	-0.0498	-0.1051	0.0471	0.0969	0.0553	0.0761	-0.0415
7(H)	0.0555	0.0093	0.0960	0.0405	0.0462	0.0434	0.0057
8(H)	0.0518	0.0151	0.1050	0.0532	0.0367	0.0449	-0.0165
9(H)	0.0550	0.0231	0.0945	0.0395	0.0319	0.0357	-0.0076
10(H)	0.0565	0.0187	0.1022	0.0457	0.0378	0.0417	-0.0080
11(O)	-0.1822	-0.2423	-0.0374	0.1448	0.0601	0.1024	-0.0847
12(N)	0.2468	0.1347	0.2636	0.0167	0.1121	0.0644	0.0954
13(O)	-0.1884	-0.3605	-0.1106	0.0779	0.1721	0.1250	0.0943
14(O)	-0.1939	-0.3501	-0.1375	0.0564	0.1562	0.1063	0.0998
15(H)	0.1382	0.1163	0.1715	0.0334	0.0219	0.0276	-0.0115

Supplementary Table 6 | The calculated adsorption energy of PMS on LFO and LFCO (121) surfaces (unit: eV)

Catalyst	E (surface + PMS)	E (surface)	E (PMS)	ΔE (ads)
LFO	-979.92	-941.70	-36.65	-1.57
LFCO-Cu	-974.68	-936.19	-36.65	-1.84
LFCO-Fe	-974.62	-936.19	-36.65	-1.78

Supplementary Table 7 | PMS adsorption bond length at different sites.

Site	ΔE (ads)	$l_{(O\text{-}O)}$ (Å)	$l_{(O\text{-}H)}$ (Å)	$l_{(S\text{-}O)}$ (Å)
PMS		1.349	1.055	1.93
LFO-Fe	-1.569	1.489	1.0480	1.775
LFCO-Fe	-1.843	1.501	1.053	1.777
LFCO-Cu	-1.785	1.500	1.077	1.723

Supplementary Table 8 | Fitting parameters for transient PL spectra of LFO and LFCO.

Catalyst	τ_1 (ns)	Rel (%)	τ_2 (ns)	Rel (%)	τ (ns)
LFO	0.23	41.28	2.82	58.72	2.15
LFCO	0.22	36.23	3.65	63.77	3.05

Supplementary Table 9 | The results of the LFO and LFCO Hall effect tests.

Catalyst	specific resistance	mobility ratio (cm^2/V.S)	carrier concentration (1/cm^3)	hall coefficient (cm^3/C)
LFO	3.90E+03	61.32	3.62E+15	−1.41E+02
LFCO	2.32E+03	70.71	4.45E+15	−2.59E+02

Supplementary Table 10 | Total metal ions detected by ICP-MS during reaction in 7 days.

Time (day)	Fe (mg/L)	Cu (mg/L)
0	0.011	0.005
1	0.012	0.004
2	0.012	0.006
3	0.012	0.006
4	0.012	0.006
5	0.014	0.006
6	0.014	0.006
7	0.014	0.006

Supplementary Table 11 | Performance and cost comparison of recently reported photocatalysts

Catalyst	k (min^{-1})	Cost (CNY)	Ref.
$B_{0.05}$-C_3N_4	0.0213	44.57	36
$Fe_3O_4/TiO_2/CuO$	0.0206	35.27	37
Ce-PDMS-PbO_2/SS	0.0270	30.89	38
PdNCs/CoAl(O)/rGO	0.0377	27.54	39
Co-$SrTiO_3$	0.0487	19.67	40
BiC-0.05	0.0139	51.72	41
$LaFe_{0.95}Cu_{0.05}O_3$	0.0790	13.72	This work
La-$SrTiO_3$	0.0412	21.19	41
Co-$Bi_2O_2CO_3$	0.0349	34.63	42
$Bi_2O_3/Bi_2O_2CO_3$	0.0213	42.91	43
$LaFeO_3$	0.0115	47.39	This work
Fe-CN	0.0642	24.66	44
$La_{0.08}MnO_{3-\sigma}$	0..0813	16.89	42
La_2CuO_4	0.0198	54.81	46
ZnxCo-ZIFs	0.0447	41.23	47

4. Supplementary Notes

4.1 Supplementary Note 1 | Identification of the TM (Cu, Ni, Fe)-N4 site

(1) Chemical structure

From BET results, the N_2 adsorption-desorption isotherm of LFO and LFCO appertained to the typical type IV curve with type 3 hysteresis loop based on the IUPAC classification. The specific surface area of LFCO (10.69 m^2 g^{-1}) is slightly increased as compared with LFO (8.09 m^2 g^{-1}), which is beneficial for adsorption (Supplementary Fig. 8)[16,17]. The similar diffraction peaks in XRD represents the successful incorporation of Cu atoms into the lattice without forming any impurity (Supplementary Fig. 9). XPS shows the presence of Cu element in XPS survey spectra as well as the new forming Cu-O peaks in O 1*s* spectra LFCO, indicating that Cu was successfully doped and formed a valence bond structure with O (Supplementary Fig. 10). Meanwhile, the Cu doping had little effect on adsorbed oxygen and oxygen vacancy and could be ignored[18,19]. In FTIR, the peak at 560 cm^{-1} and 771 cm^{-1} were assigned to Fe-O stretching vibration, and the strong octahedral coordination, respectively. The peaks at 1361 cm^{-1} and 1600 cm^{-1} correspond to the presence of water molecules adsorbed on the catalyst surface and form hydrogen bonds[20,21]. The typical characteristic band of LFCO is consistent with that of LFO excepting the new band at 482 cm^{-1} belongs to Cu-O stretching modes (Supplementary Fig. 12).

(2) Electronic structure

From the XPS Fe 2*p* spectra, Fe^{2+}/Fe^{3+} ratio increases from 0.41 to 0.62 in LFCO with the negative shift of peak position from 710.01 eV to 709.17 eV, implying a higher electron density on Fe in LFCO. The Cu 2*p* peaks in centered at about 932.70 eV and 953.90 eV indicate the valence of Cu in LFCO is mainly +1, while the La 3d peaks at about 837.95 eV and 855 eV indicate the valence of La in LFCO is mainly +3 (Supplementary Fig. 13) [16,22]. Furthermore, XPS analysis of the used and fresh LFCO catalysts show that the binding energy of Fe

$2p1/2$ level in used LFCO is lower than that of fresh LFCO, while Cu^{2+} has the opposite trend (Supplementary Fig.18). Charge transfer from partially reduced neighboring Cu atoms would increase the electron density on Fe causing the shift of binding energy. By following the redox cycle between Fe and Cu, the superior catalytic activity and outstanding stability of the LFCO catalyst perpetuated even after 7-days.

4.2 Supplementary Note 2 | Confirming the critical role of Cu-O-Fe sites in PMS activation.

(1) Identify the band position of LFCO.

The VB maximum is decreased from 1.29 eV for LFO to 0.91 eV for LFCO. The CB levels were adding the E_g values to the VB levels and are −1.07 and −1.34 eV for LFO and LFCO, respectively (Supplementary Fig. 20). UPS was used to determine the ionization potential, which is equivalent to the valence band energy (E_{VB}). According to the linear intersection method, the E_{VB} of LFO and LFCO was calculated to be 6.18 eV and 5.83 eV (vs. vacuum) from $hv + E_{Fermi} - E_{Cutoff}$ (hv of 21.22 eV: the excitation energy of the He I Source Gun). Then the E_{VB} of LFO and LFCO vs. RHE were converted to be 1.74 eV and 1.39 eV, based on the relationship between the vacuum energy (E_{vacuum}) and the RHE potential (E_{RHE}), $E_{vacuum} = E_{RHE} - 4.44$ eV. Then the E_{VB} of LFO and LFCO vs. NHE was converted to 1.32 eV and 0.97 eV from the equation $E_{(RHE)} = E_{(NHE)} + 0.0591$ pH. Combined with the bandgap values calculated from UV-vis DRS spectra and the VB XPS spectra, the band positions of LFO and LFCO could be then obtained [23]. These results show that VB position of LFCO is lower than that of OH^-/•OH (1.99 eV), proving the detected DMPO-•OH signals are from PMS activation (Supplementary Fig. 21).

(2) Charge separation and transfer

SPV is reported to be caused by the surface charge concentration originating from the photo-generated charge transferring. LFCO exhibits the higher SPV signal than LFO, indicates the effective separation and transfer of photogenerated charges (Supplementary Fig. 23). The charge separation efficiency is further investigated by

steady-state PL spectra, and a broad emission peak derived from band-to-band transition is observed at ca. 425 nm. The lower PL peak intensity of LFCO illustrates its suppressed photoinduced charge recombination that probably arises from the enhanced charge transport. More importantly, a new small peak of LFCO appeared at 543 nm, indicating that a new gap state appeared in the LFCO[24]. These results together proved that the trap states introduced by Cu doping facilitate the photoinduced charge carrier separation rather than recombination, which is consistent with the SPV results (Supplementary Fig. 24). The effect of trap states was also quantitatively reflected by TPC and the corresponding photogenerated charge density, which was calculated by integrating the measured transient photocurrent density minus the steady-state value of photocurrent with time. The higher TPC response of LFCO indicates the excellent separation of photoexcited carriers. Meanwhile, the photogenerated charge density of LFCO (21.327 $\mu C\ cm^{-2}$) is nearly 20 times that on the surface of LFO (1.039 $\mu C\ cm^{-2}$), indicating that more photogenerated electrons can participate in the catalytic reaction[25-27] (Supplementary Fig. 25). In situ XPS spectra show that the peaks of La 3*d* shift indistinctively, indicates that La hardly participates in the reaction process. The increased peak area of metal-O bonds indicates enhanced Fe/Cu-O interaction during the reaction, showing lots of electron transfer occurs in the Cu-O-Fe structure, accelerating the reaction[28,29]. We infer that the electrons are localized at Cu atoms first, then they transfer from Cu to Fe atoms and accumulate on Fe via the super-exchange effect (Supplementary Fig. 26). Then,the charge carrier lifetimes of LFO and LFCO are further evaluated by TRPL. The PL decay can be well fitted to a double-exponential model (data in Supplementary Table 8), and the LFCO manifests a longer average lifetime (3.05 ns) than LFO (2.15 ns), indicating that more long-lived photoinduced electrons and holes can participate in the reaction for LFCO[30,31] (Supplementary Fig. 27).

EIS was employed to incisively illustrate changes in the resistances and capacitances that are associated with charge trapping and transfer. It shows that the LFCO has a smaller circle radius, indicating a more efficient

interfacial conductivity compared to LFO[28]. Furthermore, the values for the charge trapping and transfer parameters are extracted from the fitted EIS plots according to the corresponding equivalent circuit (inset), in which R_1 represents the solution resistance; R_2 represents the charge transfer resistance; and CPE_1 represents the constant phase element for the electrode interface[32]. In M-S plots, the intersection of the tangent line and the abscissa represents the CB value (V vs. Ag/AgCl). M-S plots show that that of LFO and LFCO are -1.49 V and -1.64 V, respectively. Then the E_{CB} of LFO and LFCO (V vs. NHE), are converted to be -0.97 eV and -1.29 eV from the equation $E_{(NHE)} = E_{(Ag/AgCl)} + 0.197$. These results are consistent with the results calculated by the XPS, UPS, and UV-vis. Besides, as derived from the Mott-Schottky plots (Supplementary Methods 1.4), the carrier concentration of LFCO is 4.45×10^{15} cm^{-3}, which is 1.5 times that of LFCO (3.01×10^{15} cm^{-3}). This evidences the boosted charge transfer, also by other spectra results[33] (Supplementary Fig. 28).

(3) PMS activation pathway.

A combination of quenching experiments and EPR were conducted to confirm that $^1O_2$ and **$\bullet OH/SO_4^{\bullet -}$** are generated during PMS activation In the N_2 and O_2 atmosphere, the almost unchanged degradation indicates that the generation of singlet oxygen is not formed by the transformation of $\bullet O_2^-$, which can be further proved by the weak signal of DMPO-$\bullet O_2^-$. The possibility for the generation of •OH from H_2O oxidization and the evolution of $\bullet O_2^-$ to $^1O_2$ were also excluded (Supplementary Fig. 31). Then, CV and LSV curves are used to study the electron transfer behavior between PMS and LFCO. CV curves show a distinct peak at − 0.17V and increased capacitance for after adding PMS, which could be attributed to the efficient charging process between LFCO and PMS[24]. For LSV, the current density of LFCO significantly increased when adding PMS into the system, confirming the enhanced electron transfer between PMS and LFCO[34] (Supplementary Fig. 32). In situ ATR-FTIR proved the PMS adsorption and efficient decomposition on LFCO surface[23] (Supplementary Fig. 33). The characteristic vibration of SO_3 in the in situ Raman spectra appears both red-shifted (1071 cm^{-1}) and

blue-shifted (1055 cm^{-1}). This shows that the electron transfer occurs and its orientation between SO_3 in HSO_5^- and LFCO might be different (Supplementary Fig. 34).

4.3 Supplementary Note 3 | ONP degradation performance in LFCO/PMS/Vis system.

In situ UV-vis absorption spectra and HPLC results show that, the degradation rate of ONP is low in the PMS system and OAP is not generated. Under the photocatalytic condition, though the peak of ONP decreases slowly, the characteristic peaks of OAP increase first and then decrease, indicating that part of ONP is reduced to OAP during degradation. This also shows the dominance of photoreduction in the process of reducing ONP to OAP and reactive oxygen species are responsible for the subsequent ring-opening process of OAP (Supplementary Fig. 36).

Then, the role of reactive species in ONP degradation was identified. After adding $K_2Cr_2O_7$ as the quenching agent of e^-, the OAP peak at 225 nm did not appear, indicating that e^- plays a major role in the reduction of ONP to OAP. Besides, the peak intensity of the benzene ring is unchanged, indicating that electron reduction is the first important step of ONP degradation. After quenching $^1O_2$, e^- and •OH remained in the system. The concentration of OAP continued to increase and the peak intensity of the benzene ring was significantly decreased. However, after quenching •OH ($^1O_2$ and e^- remained in the system.), the peak intensity of OAP increased and then decreased, and the peak intensity of the benzene ring is slightly decreased (Supplementary Fig. 40). These results proved the reduction-oxidation coupling degradation process of ONP.

4.4 Supplementary Note 4 | Toxicity assessment and application.

In Supplementary Fig. 44a, the Oral rat LD50 values of ONP were predicted as 800.65 mg/kg, indicating that it is slightly toxic to Oral rat. The higher the LD50 value is, the lower the toxicity is. Interestingly, most reduction products (**P1**, **P2**, and **P3**) in solution have lower LD50 values than parent ONP, implying that the reduction of ONP increased their acute toxicity risk to rat. In Supplementary Fig. 44b, compounds except **P3** and **P6** have

lower predictive values of developmental toxicity than ONP. In Supplementary Fig. 44c, the mutagenicity of compounds **P1**, **P5**, and **P7** is higher than that of the parent ONP. In Supplementary Fig. 44d, except for compounds **P4** and **P6**, all compounds have lower bioaccumulation factors than ONP, indicating that these intermediates are less likely to accumulate in organisms than ONP[35]. Intermediates may have different levels of toxicity but can be effectively mineralized into CO_2 and H_2O by oxidation degradation. This implies that the co-existence of reduction and oxidization processes helps to reduce the environmental risk of the degradation products.

For application, a larger LFCO@CFC fixed bed reactor (diameter: 8 cm, high: 16 cm) was constructed for large-scale degradation of ONP. Compared to the small equipment, the scaled-up reactor volume is expanded to 16 times and the flow rate is amplified twice (adjusted to 15 L/h). The scale-up setup system can also realize high stability and durability in 7 days (Supplementary Fig. 45c). The degradation efficiency of ONP remained > 97 % in the real industrial wastewater from Shanxi Coking Coal Group Co., LTD.. Besides, ICP-MS analysis showed that the leaching concentration of Cu and Fe was negligible. With a larger reactor size, the ONP degradation rate is lower than that of the small equipment, possibly due to the uneven light intensity.

Supplementary References

1. Yang, G. *et al*. Regulating Fe-spin state by atomically dispersed Mn-N in Fe-N-C catalysts with high oxygen reduction activity. *Nat. Commun.* **12,** 1734, (2021).
2. Blochl, P. E., Projector augmented-wave method. *Phys. Rev. B. Condens. Matter* **50,** 17953-17979, (1994).
3. Delley, B., From molecules to solids with the DMol3 approach. *J. Chem. Phys.* **113,** 7756-7764, (2000).
4. John P. Perdew & K. B., Matthias Ernzerhof, Generalized gradient approximation made simple. *Phys. Rev. Lett.* **77,** 3865-3868, (1996).
5. G. Kresse, J. F., Efficiency of ab-initio total energy calculations for metals and semiconductors using a plane- wave basis set. *Comp. Mater. Sci.* **6,** 15-50, (1996).
6. Delley, B., An all-electron numerical method for solving the local density functional for polyatomic molecules. *J. Chem. Phys.* **92,** 508-517, (1990).
7. Lu, T. & Chen, F., Multiwfn: a multifunctional wavefunction analyzer. *J. Comput. Chem.* **33,** 580-592, (2012).
8. G. Kresse, D. J., From ultrasoft pseudopotentials to the projector augmented-wave method. *Phys. Rev. B.* **59,** 1758-1775, (1999).
9. Zhang, R. *et al*. Kinetics and modeling of sulfonamide antibiotic degradation in wastewater and human urine by UV/H_2O_2 and UV/PDS. *Water Res.* **103,** 283-292, (2016).
10. Antonopoulou *et al*., An overview of homogeneous and heterogeneous photocatalysis applications for the removal of pharmaceutical compounds from real or synthetic hospital wastewaters under lab or pilot scale. *Sci. Total Environ.* **765**, 144163, (2021).
11. Spasiano, D. *et al*., Solar photocatalysis: Materials, reactors, some commercial, and pre-industrialized applications. A comprehensive approach. *Appl Cata. B: Environ.* **170-171**, 90-123, (2015).

12. Sixto, M. *et al.*, Photocatalysis with solar energy at a pilot-plant scale: an overview. *Appl Cata. B: Environ.* **37**, 1-15, (2002).

13. Zhu, B. *et al.*, In situ growth g-C_3N_4 particles on carbon fiber cloth as flexible and easily reusable visible-light-driven photocatalysts. *Mater. Lett.* **335**,133744, (2023).

14. Katz, M. B. *et al.* Self-regeneration of Pd-$LaFeO_3$ catalysts: new insight from atomic-resolution electron microscopy. *J. Am. Chem. Soc.* **133,** 18090-18093, (2011).

15. Pan, K. *et al.* Oxygen vacancy mediated surface charge redistribution of Cu-substituted $LaFeO_3$ for degradation of bisphenol A by efficient decomposition of H_2O_2. *J. Hazard. Mater.* **389,** 122072, (2020).

16. Zhang, X. *et al.* FeO_6 octahedral distortion activates lattice xxygen in perovskite ferrite for methane partial xxidation coupled with CO_2 splitting. *J. Am. Chem. Soc.* **142,** 11540-11549, (2020).

17. Nie, Y., Zhang, L., Li, Y. Y. & Hu, C., Enhanced Fenton-like degradation of refractory organic compounds by surface complex formation of $LaFeO_3$ and H_2O_2. *J. Hazard. Mater.* **294,** 195-200, (2015).

18. Nguyen, T. X. *et al.* Advanced high entropy perovskite oxide electrocatalyst for oxygen evolution reaction. *Adv. Funct. Mater.* **31,** 2101632, (2021).

19. Yang, W. T. *et al.* High-performance and long-term stability of mesoporous Cu-doped TiO_2 microsphere for catalytic CO oxidation. *J. Hazard. Mater.* **403,** 123630, (2021).

20. Ashokkumar, M. & Muthukumaran, S., Microstructure, optical and FTIR studies of Ni, Cu, co-doped ZnO nanoparticles by co-precipitation method. *Opt. Mater.* **37,** 671-678, (2014).

21. Jawad, A. *et al.* Tuning of persulfate activation from a free radical to a nonradical pathway through the incorporation of non-redox magnesium oxide. *Environ. Sci. Technol.* **54,** 2476-2488, (2020).

22. Zhang, J. *et al.* Electrospun $CuAl_2O_4$ hollow nanofibers as visible light photocatalyst with enhanced activity and excellent stability under acid and alkali conditions. *Cryst. Eng. Comm.* **20,** 312-322, (2018).

23. Gao, Y. *et al*. Electronic structure modulation of graphitic carbon nitride by oxygen doping for enhanced catalytic degradation of organic pollutants through peroxymonosulfate activation. *Environ. Sci. Technol.* **52,** 14371-14380, (2018).

24. Zhao, D. *et al*. Boron-doped nitrogen-deficient carbon nitride-based Z-scheme heterostructures for photo-catalytic overall water splitting. *Nat. Energy.* **6,** 388-397, (2021).

25. Ren, T. *et al*. Highly efficient and stable p-$LaFeO_3$/n-ZnO heterojunction photocatalyst for phenol degradation under visible light irradiation. *J. Hazard. Mater.* **377,** 195-205, (2019).

26. Gao, R. T. *et al*. Single-atomic-site platinum steers photogenerated charge carrier lifetime of hematite nanoflakes for photoelectrochemical water splitting. *Nat. Commun.* **14,** 2640, (2023).

27. Zhou, W. *et al*. Enhanced photocatalytic degradation of xylene by blackening TiO_2 nanoparticles with high dispersion of CuO. *J. Hazard. Mater.* **391,** 121642, (2020).

28. Yang, J., Jing, J. & Zhu, Y., A full-spectrum porphyrin-fullerene D-A supramolecular photocatalyst with Giant Built-in electric field for efficient hydrogen production. *Adv. Mater.* **33,** e2101026, (2021).

29. Zhang, Y. *et al*. Create a strong internal electric-field on PDI photocatalysts for boosting phenols degradation via preferentially exposing π-conjugated planes up to 100%. *Appl Cata. B: Environ.* **300,** 120762, (2022)

30. Jones, T. W. *et al*. Lattice strain causes non-radiative losses in halide perovskites. *Energ. Environ. Sci.* **12,** 596-606, (2019).

31. Li, Y. *et al*. Exciton-mediated energy transfer in heterojunction enables infrared light photocatalysis. *Angew. Chem. Int. Ed.* **60,** 12891-12896, (2021).

32. Yu, F. *et al*. Hierarchically porous metal-organic framework/MoS_2 interface for selective photocatalytic conversion of CO_2 with H_2O into CH_3COOH. *Angew. Chem. Int. Ed.* **60,** 24849-24853, (2021).

33. Wang, P. *et al*. Unraveling the interfacial charge migration pathway at the atomic level in a highly efficient Z-Scheme photocatalyst. *Angew. Chem. Int. Ed.* **58,** 11329-11334, (2019).

34. Liu, X. *et al*. In Situ Modulation of A-site vacancies in $LaMnO_{3.15}$ perovskite for surface lattice oxygen activation and boosted redox reactions. *Angew. Chem. Int. Ed.* **60,** 26747-26754, (2021).

35. Liu, T. *et al*. The role of reactive oxygen species and carbonate radical in oxcarbazepine degradation via UV, UV/H_2O_2: Kinetics, mechanisms and toxicity evaluation. *Water Res.* **147,** 204-213, (2018).

36. Zhan, H. *et al*. Photocatalytic O_2 activation and reactive oxygen species evolution by surface B-N bond for organic pollutants degradation. *Appl Cata. B: Environ.* **310**, 121329, (2022).

37. Kianfar, A. H.; Arayesh, M. A. Synthesis, characterization and investigation of photocatalytic and catalytic applications of Fe_3O_4/TiO_2/CuO nanoparticles for degradation of MB and reduction of nitrophenols. J. *Environ. Chem. Eng.* **8**, 103640, (2020).

38. Li, H. *et al*. Ultrasonic-electrodeposition construction of high hydrophobic Ce-PDMS-PbO_2/SS electrode for p-nitrophenol degradation: Catalytic, kinetics and mechanism. *Appl Cata. B: Environ.* **335**, 122884, (2023).

39. Zhou, Q. *et al*. Novel hierarchical carbon quantum dots-decorated BiOCl nanosheet/carbonized eggshell membrane composites for improved removal of organic contaminants from water via synergistic adsorption and photocatalysis. *Chem. Eng. J.* **420**, 129582, (2021).

40. Wang, Q. *et al*. Hierarchical-Structured Pd Nanoclusters Catalysts x-PdNCs/CoAl(O)/rGO-T by the Captopril-Capped Pd Cluster Precursor Method for the Highly Efficient 4-Nitrophenol Reduction. *ACS Appl. Mater. Interfaces*, **14**, 27775-27790, (2022).

41. Zhang, D. *et al*. Dynamic active-site induced by host-guest interactions boost the Fenton-like reaction for organic wastewater treatment. *Nat. Commun.* **14**, 3538, (2023).

42. Zhao, D.-X.; Lu, G.-P.; Cai, C. Efficient visible-light-driven Suzuki coupling reaction over Co-doped BiOCl/Ce-doped Bi2O_2CO_3 composites. *Green Chem.* **23**, 1823-1833, (2021).

43. Huang, Y. *et al*. Visible light $Bi_2S_3/Bi_2O_3/Bi_2O_2CO_3$ photocatalyst for effective degradation of organic pollutions. *Appl Cata. B: Environ.* **185**, 68-76, (2016).

44. Liu, X. *et al*. In Situ Modulation of A-Site Vacancies in $LaMnO_{3.15}$ Perovskite for Surface Lattice Oxygen Activation and Boosted Redox Reactions. *Angew. Chem. Int. Ed.* **60**, 26747-26754, (2021).

45. Chen, H. *et al*. Understanding oxygen-deficient $La_2CuO_{4-\delta}$ perovskite activated peroxymonosulfate for bisphenol A degradation: The role of localized electron within oxygen vacancy. *Appl Cata. B: Environ.* **284**, 119732, (2021).

46. Chen, Z. *et al*. Single-atom Mo-Co catalyst with low biotoxicity for sustainable degradation of high-ionization-potential organic pollutants. *Proc. Natl. Acad. Sci.* **120**, e2305933120, (2023).

47. Tang, Y. *et al.* Engineering magnetic N-doped porous carbon with super- high ciprofloxacin adsorption. capacity and wide pH adaptability. *J. Hazard. Mater*. 388, 122059, (2020).
