# Supplementary material for: Efficient reduction-oxidation coupling degradation of nitroaromatic compounds in continuous flow processes
Source: Nat Commun. 2024 Jul 29;15:6364. doi: 10.1038/s41467-024-50238-8 (PMC11286756; doi:10.1038/s41467-024-50238-8)
Supplement: Supplementary file 3 — Description of Additional Supplementary Files [file 41467_2024_50238_MOESM3_ESM.pdf]

Description of Additional Supplementary Files

File Name: Supplementary Data 1
Description: Atomic coordinates of the optimized computational models data.
