# Supplementary material for: Efficient reduction-oxidation coupling degradation of nitroaromatic compounds in continuous flow processes
Source: Nat Commun. 2024 Jul 29;15:6364. doi: 10.1038/s41467-024-50238-8 (PMC11286756; doi:10.1038/s41467-024-50238-8)
Supplement: Supplementary file 4 — Supplementary Data 1 [file 41467_2024_50238_MOESM4_ESM.pdf]

1. LCFO-121-surface

```
data_created_by_vaspkit_code
_audit_creation_date         Mon Feb 13 20:23:29 2023
_pd_phase_name                    'CIF files'
_cell_length_a                   10.88689995
_cell_length_b                   15.36690044
_cell_length_c                   22.85919952
_cell_angle_alpha                    90.0000
_cell_angle_beta                     90.0000
_cell_angle_gamma                    90.0632
_symmetry_space_group_name_H-M          'P 1'
_symmetry_Int_Tables_number          1
loop_
_symmetry_equiv_pos_as_xyz
    'x, y, z'
loop_
   _atom_site_label
   _atom_site_occupancy
   _atom_site_fract_x
   _atom_site_fract_y
   _atom_site_fract_z
   _atom_site_thermal_displace_type
   _atom_site_U_iso_or_equiv
   _atom_site_type_symbol
    O001  1.0    0.844420    0.001100    0.053340  Uiso   1.00   O
    O002  1.0    0.098682    0.250430    0.172100  Uiso   1.00   O
    O003  1.0    0.359204   -0.001276    0.300141  Uiso   1.00   O
    O004  1.0    0.020010    0.392320    0.104120  Uiso   1.00   O
    O005  1.0    0.266793    0.137577    0.220987  Uiso   1.00   O
    O006  1.0    0.555429    0.378855    0.344923  Uiso   1.00   O
    O007  1.0    0.376880    0.001110    0.068860  Uiso   1.00   O
    O008  1.0    0.625857    0.250821    0.188875  Uiso   1.00   O
    O009  1.0    0.875616    0.000879    0.307548  Uiso   1.00   O
    O010  1.0    0.519980    0.359740    0.104100  Uiso   1.00   O
    O011  1.0    0.765500    0.111478    0.220455  Uiso   1.00   O
    O012  1.0    0.036728    0.371178    0.337428  Uiso   1.00   O
    O013  1.0    0.154992    0.000826    0.175528  Uiso   1.00   O
    O014  1.0    0.911700    0.251140    0.052340  Uiso   1.00   O
    O015  1.0    0.396634    0.242359    0.297180  Uiso   1.00   O
    O016  1.0    0.736100    0.359910    0.001560  Uiso   1.00   O
    O017  1.0    0.985150    0.109710    0.120630  Uiso   1.00   O
    O018  1.0    0.239158    0.363454    0.245618  Uiso   1.00   O
    O019  1.0    0.379240    0.251120    0.036820  Uiso   1.00   O
    O020  1.0    0.628290    0.000930    0.155880  Uiso   1.00   O
    O021  1.0    0.877399    0.251148    0.288489  Uiso   1.00   O
    O022  1.0    0.236140    0.392490    0.001580  Uiso   1.00   O
    O023  1.0    0.485190    0.142300    0.120640  Uiso   1.00   O
    O024  1.0    0.742571    0.386804    0.247260  Uiso   1.00   O
    O025  1.0    0.522460    0.112150    0.002360  Uiso   1.00   O
    O026  1.0    0.010863    0.117323    0.247955  Uiso   1.00   O
    O027  1.0    0.771500    0.361960    0.121420  Uiso   1.00   O
    O028  1.0    0.017530    0.107210    0.000000  Uiso   1.00   O
    O029  1.0    0.266580    0.357010    0.119060  Uiso   1.00   O
    O030  1.0    0.509746    0.109600    0.246514  Uiso   1.00   O
    O031  1.0    0.233660    0.140080    0.103330  Uiso   1.00   O
```

O032	1.0	0.494596	0.389907	0.221174	Uiso	1.00	O
O033	1.0	0.718604	0.131160	0.339125	Uiso	1.00	O
O034	1.0	0.738590	0.145020	0.105680	Uiso	1.00	O
O035	1.0	0.212516	0.127612	0.338129	Uiso	1.00	O
O036	1.0	0.987430	0.388412	0.220476	Uiso	1.00	O
O037	1.0	0.844420	0.501100	0.053340	Uiso	1.00	O
O038	1.0	0.100030	0.750235	0.173772	Uiso	1.00	O
O039	1.0	0.359192	0.503610	0.295990	Uiso	1.00	O
O040	1.0	0.020010	0.892320	0.104120	Uiso	1.00	O
O041	1.0	0.266315	0.639324	0.220967	Uiso	1.00	O
O042	1.0	0.539629	0.879453	0.339820	Uiso	1.00	O
O043	1.0	0.376880	0.501110	0.068860	Uiso	1.00	O
O044	1.0	0.625597	0.751071	0.188368	Uiso	1.00	O
O045	1.0	0.875380	0.500103	0.308089	Uiso	1.00	O
O046	1.0	0.519980	0.859740	0.104100	Uiso	1.00	O
O047	1.0	0.766409	0.613296	0.220148	Uiso	1.00	O
O048	1.0	0.035792	0.871114	0.337598	Uiso	1.00	O
O049	1.0	0.156136	0.500819	0.175551	Uiso	1.00	O
O050	1.0	0.911700	0.751140	0.052340	Uiso	1.00	O
O051	1.0	0.395282	0.749017	0.297190	Uiso	1.00	O
O052	1.0	0.736100	0.859910	0.001560	Uiso	1.00	O
O053	1.0	0.985150	0.609710	0.120630	Uiso	1.00	O
O054	1.0	0.243330	0.865669	0.248622	Uiso	1.00	O
O055	1.0	0.379240	0.751120	0.036820	Uiso	1.00	O
O056	1.0	0.628290	0.500930	0.155880	Uiso	1.00	O
O057	1.0	0.875265	0.750479	0.288493	Uiso	1.00	O
O058	1.0	0.236140	0.892490	0.001580	Uiso	1.00	O
O059	1.0	0.485190	0.642300	0.120640	Uiso	1.00	O
O060	1.0	0.741061	0.886577	0.247762	Uiso	1.00	O
O061	1.0	0.522460	0.612150	0.002360	Uiso	1.00	O
O062	1.0	0.011022	0.615912	0.248371	Uiso	1.00	O
O063	1.0	0.771500	0.861960	0.121420	Uiso	1.00	O
O064	1.0	0.017530	0.607210	0.000000	Uiso	1.00	O
O065	1.0	0.266580	0.857010	0.119060	Uiso	1.00	O
O066	1.0	0.510158	0.615195	0.246795	Uiso	1.00	O
O067	1.0	0.233660	0.640080	0.103330	Uiso	1.00	O
O068	1.0	0.488438	0.889492	0.221700	Uiso	1.00	O
O069	1.0	0.717792	0.630443	0.338969	Uiso	1.00	O
O070	1.0	0.738590	0.645020	0.105680	Uiso	1.00	O
O071	1.0	0.213521	0.629993	0.337766	Uiso	1.00	O
O072	1.0	0.987191	0.887690	0.220986	Uiso	1.00	O
La001	1.0	0.134220	0.259760	0.055790	Uiso	1.00	La
La002	1.0	0.383184	0.010560	0.177408	Uiso	1.00	La
La003	1.0	0.626514	0.257444	0.298975	Uiso	1.00	La
La004	1.0	0.619450	0.244940	0.048720	Uiso	1.00	La
La005	1.0	0.121305	0.247836	0.292018	Uiso	1.00	La
La006	1.0	0.868490	0.494740	0.167790	Uiso	1.00	La
La007	1.0	0.621900	0.492480	0.049900	Uiso	1.00	La
La008	1.0	0.870940	0.242280	0.168960	Uiso	1.00	La
La009	1.0	0.129108	0.495520	0.294586	Uiso	1.00	La
La010	1.0	0.136670	0.007290	0.056960	Uiso	1.00	La
La011	1.0	0.384932	0.259437	0.180786	Uiso	1.00	La
La012	1.0	0.632346	0.002948	0.299484	Uiso	1.00	La
La013	1.0	0.134220	0.759760	0.055790	Uiso	1.00	La

La014	1.0	0.383891	0.507645	0.179363	Uiso	1.00	La
La015	1.0	0.629708	0.752411	0.296513	Uiso	1.00	La
La016	1.0	0.619450	0.744940	0.048720	Uiso	1.00	La
La017	1.0	0.117849	0.746718	0.291663	Uiso	1.00	La
La018	1.0	0.868490	0.994740	0.167790	Uiso	1.00	La
La019	1.0	0.621900	0.992480	0.049900	Uiso	1.00	La
La020	1.0	0.870940	0.742280	0.168960	Uiso	1.00	La
La021	1.0	0.124806	0.996592	0.294868	Uiso	1.00	La
La022	1.0	0.136670	0.507290	0.056960	Uiso	1.00	La
La023	1.0	0.383858	0.756504	0.177547	Uiso	1.00	La
La024	1.0	0.626108	0.498313	0.300829	Uiso	1.00	La
Fe001	1.0	0.378060	0.126120	0.052840	Uiso	1.00	Fe
Fe002	1.0	0.631843	0.374923	0.173688	Uiso	1.00	Fe
Fe003	1.0	0.861807	0.124281	0.294614	Uiso	1.00	Fe
Fe004	1.0	0.378060	0.376120	0.052840	Uiso	1.00	Fe
Fe005	1.0	0.622906	0.126689	0.173630	Uiso	1.00	Fe
Fe006	1.0	0.891733	0.378380	0.293956	Uiso	1.00	Fe
Fe007	1.0	0.123128	0.125166	0.173941	Uiso	1.00	Fe
Fe008	1.0	0.878060	0.376120	0.052840	Uiso	1.00	Fe
Fe009	1.0	0.878060	0.126120	0.052840	Uiso	1.00	Fe
Fe010	1.0	0.130054	0.375547	0.173829	Uiso	1.00	Fe
Fe011	1.0	0.359357	0.123520	0.296953	Uiso	1.00	Fe
Fe012	1.0	0.378060	0.626120	0.052840	Uiso	1.00	Fe
Fe013	1.0	0.630575	0.875245	0.173864	Uiso	1.00	Fe
Fe014	1.0	0.860913	0.621734	0.294736	Uiso	1.00	Fe
Fe015	1.0	0.378060	0.876120	0.052840	Uiso	1.00	Fe
Fe016	1.0	0.623117	0.626495	0.173765	Uiso	1.00	Fe
Fe017	1.0	0.890785	0.878149	0.294124	Uiso	1.00	Fe
Fe018	1.0	0.123239	0.624921	0.174113	Uiso	1.00	Fe
Fe019	1.0	0.878060	0.876120	0.052840	Uiso	1.00	Fe
Fe020	1.0	0.393174	0.875050	0.296687	Uiso	1.00	Fe
Fe021	1.0	0.878060	0.626120	0.052840	Uiso	1.00	Fe
Fe022	1.0	0.130437	0.875589	0.174448	Uiso	1.00	Fe
Fe023	1.0	0.359779	0.625797	0.296077	Uiso	1.00	Fe
Cu001	1.0	0.388202	0.373671	0.304391	Uiso	1.00	Cu

2. LCFO-Cu-PMS

```
data_created_by_vaspkit_code
_audit_creation_date         Tue Feb 14 14:24:02 2023
_pd_phase_name                     'CIF files'
_cell_length_a                    10.88689995
_cell_length_b                    15.36690044
_cell_length_c                    22.85919952
_cell_angle_alpha                     90.0000
_cell_angle_beta                      90.0000
_cell_angle_gamma                     90.0632
_symmetry_space_group_name_H-M          'P 1'
_symmetry_Int_Tables_number           1
loop_
_symmetry_equiv_pos_as_xyz
    'x, y, z'
loop_
   _atom_site_label
   _atom_site_occupancy
   _atom_site_fract_x
   _atom_site_fract_y
   _atom_site_fract_z
   _atom_site_thermal_displace_type
   _atom_site_U_iso_or_equiv
   _atom_site_type_symbol
    O001  1.0    0.844420    0.001100    0.053340  Uiso   1.00   O
    O002  1.0    0.097608    0.250239    0.170161  Uiso   1.00   O
    O003  1.0    0.359304    0.997955    0.300155  Uiso   1.00   O
    O004  1.0    0.020010    0.392320    0.104120  Uiso   1.00   O
    O005  1.0    0.267631    0.136198    0.219998  Uiso   1.00   O
    O006  1.0    0.573677    0.379152    0.347821  Uiso   1.00   O
    O007  1.0    0.376880    0.001110    0.068860  Uiso   1.00   O
    O008  1.0    0.624637    0.249337    0.189640  Uiso   1.00   O
    O009  1.0    0.877396    0.999635    0.305355  Uiso   1.00   O
    O010  1.0    0.519980    0.359740    0.104100  Uiso   1.00   O
    O011  1.0    0.766257    0.110563    0.220148  Uiso   1.00   O
    O012  1.0    0.038632    0.369392    0.335990  Uiso   1.00   O
    O013  1.0    0.153381    0.000222    0.174843  Uiso   1.00   O
    O014  1.0    0.911700    0.251140    0.052340  Uiso   1.00   O
    O015  1.0    0.385906    0.245262    0.292924  Uiso   1.00   O
    O016  1.0    0.736100    0.359910    0.001560  Uiso   1.00   O
    O017  1.0    0.985150    0.109710    0.120630  Uiso   1.00   O
    O018  1.0    0.230622    0.363847    0.237807  Uiso   1.00   O
    O019  1.0    0.379240    0.251120    0.036820  Uiso   1.00   O
    O020  1.0    0.628290    0.000930    0.155880  Uiso   1.00   O
    O021  1.0    0.878318    0.249861    0.288068  Uiso   1.00   O
    O022  1.0    0.236140    0.392490    0.001580  Uiso   1.00   O
    O023  1.0    0.485190    0.142300    0.120640  Uiso   1.00   O
    O024  1.0    0.744579    0.384788    0.245630  Uiso   1.00   O
    O025  1.0    0.522460    0.112150    0.002360  Uiso   1.00   O
    O026  1.0    0.011969    0.117110    0.247032  Uiso   1.00   O
    O027  1.0    0.771500    0.361960    0.121420  Uiso   1.00   O
    O028  1.0    0.017530    0.107210    0.000000  Uiso   1.00   O
    O029  1.0    0.266580    0.357010    0.119060  Uiso   1.00   O
    O030  1.0    0.510489    0.108393    0.249358  Uiso   1.00   O
    O031  1.0    0.233660    0.140080    0.103330  Uiso   1.00   O
```

O032	1.0	0.496779	0.389297	0.223599	Uiso	1.00	O
O033	1.0	0.725475	0.128407	0.340031	Uiso	1.00	O
O034	1.0	0.738590	0.145020	0.105680	Uiso	1.00	O
O035	1.0	0.211158	0.123986	0.335436	Uiso	1.00	O
O036	1.0	0.985628	0.386037	0.218585	Uiso	1.00	O
O037	1.0	0.844420	0.501100	0.053340	Uiso	1.00	O
O038	1.0	0.100490	0.749752	0.173561	Uiso	1.00	O
O039	1.0	0.358659	0.501504	0.295239	Uiso	1.00	O
O040	1.0	0.020010	0.892320	0.104120	Uiso	1.00	O
O041	1.0	0.266465	0.637124	0.220776	Uiso	1.00	O
O042	1.0	0.540498	0.876667	0.339752	Uiso	1.00	O
O043	1.0	0.376880	0.501110	0.068860	Uiso	1.00	O
O044	1.0	0.624940	0.750607	0.187862	Uiso	1.00	O
O045	1.0	0.876193	0.498090	0.305910	Uiso	1.00	O
O046	1.0	0.519980	0.859740	0.104100	Uiso	1.00	O
O047	1.0	0.766583	0.613493	0.219950	Uiso	1.00	O
O048	1.0	0.034658	0.869272	0.337125	Uiso	1.00	O
O049	1.0	0.154409	0.499728	0.173376	Uiso	1.00	O
O050	1.0	0.911700	0.751140	0.052340	Uiso	1.00	O
O051	1.0	0.393123	0.747033	0.296349	Uiso	1.00	O
O052	1.0	0.736100	0.859910	0.001560	Uiso	1.00	O
O053	1.0	0.985150	0.609710	0.120630	Uiso	1.00	O
O054	1.0	0.240461	0.864754	0.248158	Uiso	1.00	O
O055	1.0	0.379240	0.751120	0.036820	Uiso	1.00	O
O056	1.0	0.628290	0.500930	0.155880	Uiso	1.00	O
O057	1.0	0.876800	0.748771	0.287827	Uiso	1.00	O
O058	1.0	0.236140	0.892490	0.001580	Uiso	1.00	O
O059	1.0	0.485190	0.642300	0.120640	Uiso	1.00	O
O060	1.0	0.739582	0.884303	0.248650	Uiso	1.00	O
O061	1.0	0.522460	0.612150	0.002360	Uiso	1.00	O
O062	1.0	0.012677	0.614452	0.248390	Uiso	1.00	O
O063	1.0	0.771500	0.861960	0.121420	Uiso	1.00	O
O064	1.0	0.017530	0.607210	0.000000	Uiso	1.00	O
O065	1.0	0.266580	0.857010	0.119060	Uiso	1.00	O
O066	1.0	0.510593	0.614440	0.247169	Uiso	1.00	O
O067	1.0	0.233660	0.640080	0.103330	Uiso	1.00	O
O068	1.0	0.486693	0.888655	0.221594	Uiso	1.00	O
O069	1.0	0.721809	0.629589	0.338728	Uiso	1.00	O
O070	1.0	0.738590	0.645020	0.105680	Uiso	1.00	O
O071	1.0	0.215355	0.628221	0.337822	Uiso	1.00	O
O072	1.0	0.985985	0.886133	0.220006	Uiso	1.00	O
O073	1.0	0.461999	0.202852	0.412475	Uiso	1.00	O
O074	1.0	0.254457	0.226112	0.452255	Uiso	1.00	O
O075	1.0	0.437810	0.238463	0.517228	Uiso	1.00	O
O076	1.0	0.390970	0.352941	0.446481	Uiso	1.00	O
O077	1.0	0.294735	0.371797	0.401948	Uiso	1.00	O
La001	1.0	0.134220	0.259760	0.055790	Uiso	1.00	La
La002	1.0	0.383304	0.010646	0.177805	Uiso	1.00	La
La003	1.0	0.617224	0.253013	0.307459	Uiso	1.00	La
La004	1.0	0.619450	0.244940	0.048720	Uiso	1.00	La
La005	1.0	0.125489	0.247202	0.291250	Uiso	1.00	La
La006	1.0	0.868490	0.494740	0.167790	Uiso	1.00	La
La007	1.0	0.621900	0.492480	0.049900	Uiso	1.00	La
La008	1.0	0.870940	0.242280	0.168960	Uiso	1.00	La

La009	1.0	0.131662	0.493319	0.295357	Uiso	1.00	La
La010	1.0	0.136670	0.007290	0.056960	Uiso	1.00	La
La011	1.0	0.384922	0.260584	0.182430	Uiso	1.00	La
La012	1.0	0.633423	0.001113	0.301849	Uiso	1.00	La
La013	1.0	0.134220	0.759760	0.055790	Uiso	1.00	La
La014	1.0	0.383160	0.503793	0.180526	Uiso	1.00	La
La015	1.0	0.629517	0.750145	0.296586	Uiso	1.00	La
La016	1.0	0.619450	0.744940	0.048720	Uiso	1.00	La
La017	1.0	0.119915	0.745020	0.291956	Uiso	1.00	La
La018	1.0	0.868490	0.994740	0.167790	Uiso	1.00	La
La019	1.0	0.621900	0.992480	0.049900	Uiso	1.00	La
La020	1.0	0.870940	0.742280	0.168960	Uiso	1.00	La
La021	1.0	0.124203	0.995202	0.294669	Uiso	1.00	La
La022	1.0	0.136670	0.507290	0.056960	Uiso	1.00	La
La023	1.0	0.382663	0.755122	0.177549	Uiso	1.00	La
La024	1.0	0.630191	0.495860	0.300697	Uiso	1.00	La
Fe001	1.0	0.378060	0.126120	0.052840	Uiso	1.00	Fe
Fe002	1.0	0.632609	0.373335	0.175292	Uiso	1.00	Fe
Fe003	1.0	0.863332	0.123833	0.293741	Uiso	1.00	Fe
Fe004	1.0	0.378060	0.376120	0.052840	Uiso	1.00	Fe
Fe005	1.0	0.622871	0.127054	0.174171	Uiso	1.00	Fe
Fe006	1.0	0.892299	0.375751	0.292596	Uiso	1.00	Fe
Fe007	1.0	0.123637	0.125068	0.173477	Uiso	1.00	Fe
Fe008	1.0	0.878060	0.376120	0.052840	Uiso	1.00	Fe
Fe009	1.0	0.878060	0.126120	0.052840	Uiso	1.00	Fe
Fe010	1.0	0.127303	0.375071	0.172051	Uiso	1.00	Fe
Fe011	1.0	0.359442	0.123713	0.297911	Uiso	1.00	Fe
Fe012	1.0	0.378060	0.626120	0.052840	Uiso	1.00	Fe
Fe013	1.0	0.629675	0.874903	0.173963	Uiso	1.00	Fe
Fe014	1.0	0.862823	0.620426	0.294233	Uiso	1.00	Fe
Fe015	1.0	0.378060	0.876120	0.052840	Uiso	1.00	Fe
Fe016	1.0	0.622560	0.626202	0.173814	Uiso	1.00	Fe
Fe017	1.0	0.890953	0.876146	0.293635	Uiso	1.00	Fe
Fe018	1.0	0.123281	0.623825	0.173848	Uiso	1.00	Fe
Fe019	1.0	0.878060	0.876120	0.052840	Uiso	1.00	Fe
Fe020	1.0	0.393461	0.874499	0.296329	Uiso	1.00	Fe
Fe021	1.0	0.878060	0.626120	0.052840	Uiso	1.00	Fe
Fe022	1.0	0.129745	0.875098	0.174056	Uiso	1.00	Fe
Fe023	1.0	0.360653	0.623381	0.296098	Uiso	1.00	Fe
Cu001	1.0	0.391298	0.374040	0.318171	Uiso	1.00	Cu
S001	1.0	0.386943	0.242484	0.459180	Uiso	1.00	S
H001	1.0	0.234201	0.325508	0.415437	Uiso	1.00	H

3. LCFO-Fe-PMS

```
data_created_by_vaspkit_code
_audit_creation_date         Mon Feb 13 20:24:25 2023
_pd_phase_name                    'CIF files'
_cell_length_a                   10.88689995
_cell_length_b                   15.36690044
_cell_length_c                   22.85919952
_cell_angle_alpha                    90.0000
_cell_angle_beta                     90.0000
_cell_angle_gamma                    90.0632
_symmetry_space_group_name_H-M           'P 1'
_symmetry_Int_Tables_number          1
loop_
_symmetry_equiv_pos_as_xyz
    'x, y, z'
loop_
   _atom_site_label
   _atom_site_occupancy
   _atom_site_fract_x
   _atom_site_fract_y
   _atom_site_fract_z
   _atom_site_thermal_displace_type
   _atom_site_U_iso_or_equiv
   _atom_site_type_symbol
    O001  1.0    0.844420    0.001100    0.053340  Uiso   1.00   O
    O002  1.0    0.098641    0.249859    0.171685  Uiso   1.00   O
    O003  1.0    0.360389    0.998474    0.299978  Uiso   1.00   O
    O004  1.0    0.020010    0.392320    0.104120  Uiso   1.00   O
    O005  1.0    0.266571    0.137016    0.220856  Uiso   1.00   O
    O006  1.0    0.549644    0.373368    0.346476  Uiso   1.00   O
    O007  1.0    0.376880    0.001110    0.068860  Uiso   1.00   O
    O008  1.0    0.626309    0.250754    0.188658  Uiso   1.00   O
    O009  1.0    0.874329    0.000061    0.305879  Uiso   1.00   O
    O010  1.0    0.519980    0.359740    0.104100  Uiso   1.00   O
    O011  1.0    0.765853    0.111466    0.219819  Uiso   1.00   O
    O012  1.0    0.032636    0.369647    0.337240  Uiso   1.00   O
    O013  1.0    0.154624    0.000380    0.175382  Uiso   1.00   O
    O014  1.0    0.911700    0.251140    0.052340  Uiso   1.00   O
    O015  1.0    0.395098    0.241039    0.296532  Uiso   1.00   O
    O016  1.0    0.736100    0.359910    0.001560  Uiso   1.00   O
    O017  1.0    0.985150    0.109710    0.120630  Uiso   1.00   O
    O018  1.0    0.237347    0.361469    0.245941  Uiso   1.00   O
    O019  1.0    0.379240    0.251120    0.036820  Uiso   1.00   O
    O020  1.0    0.628290    0.000930    0.155880  Uiso   1.00   O
    O021  1.0    0.874977    0.250286    0.288053  Uiso   1.00   O
    O022  1.0    0.236140    0.392490    0.001580  Uiso   1.00   O
    O023  1.0    0.485190    0.142300    0.120640  Uiso   1.00   O
    O024  1.0    0.738010    0.388427    0.250668  Uiso   1.00   O
    O025  1.0    0.522460    0.112150    0.002360  Uiso   1.00   O
    O026  1.0    0.010997    0.117000    0.248158  Uiso   1.00   O
    O027  1.0    0.771500    0.361960    0.121420  Uiso   1.00   O
    O028  1.0    0.017530    0.107210    0.000000  Uiso   1.00   O
    O029  1.0    0.266580    0.357010    0.119060  Uiso   1.00   O
    O030  1.0    0.509636    0.109044    0.246079  Uiso   1.00   O
    O031  1.0    0.233660    0.140080    0.103330  Uiso   1.00   O
```

O032	1.0	0.494008	0.388033	0.221488	Uiso	1.00	O
O033	1.0	0.718671	0.129008	0.338655	Uiso	1.00	O
O034	1.0	0.738590	0.145020	0.105680	Uiso	1.00	O
O035	1.0	0.212964	0.126664	0.338000	Uiso	1.00	O
O036	1.0	0.984411	0.386063	0.220004	Uiso	1.00	O
O037	1.0	0.844420	0.501100	0.053340	Uiso	1.00	O
O038	1.0	0.101082	0.750362	0.174203	Uiso	1.00	O
O039	1.0	0.357657	0.500452	0.293043	Uiso	1.00	O
O040	1.0	0.020010	0.892320	0.104120	Uiso	1.00	O
O041	1.0	0.268990	0.637607	0.220611	Uiso	1.00	O
O042	1.0	0.537268	0.878007	0.338817	Uiso	1.00	O
O043	1.0	0.376880	0.501110	0.068860	Uiso	1.00	O
O044	1.0	0.624531	0.750338	0.188145	Uiso	1.00	O
O045	1.0	0.876499	0.500310	0.308113	Uiso	1.00	O
O046	1.0	0.519980	0.859740	0.104100	Uiso	1.00	O
O047	1.0	0.767700	0.614862	0.219696	Uiso	1.00	O
O048	1.0	0.032777	0.871770	0.337790	Uiso	1.00	O
O049	1.0	0.155107	0.499761	0.175349	Uiso	1.00	O
O050	1.0	0.911700	0.751140	0.052340	Uiso	1.00	O
O051	1.0	0.393224	0.748991	0.295983	Uiso	1.00	O
O052	1.0	0.736100	0.859910	0.001560	Uiso	1.00	O
O053	1.0	0.985150	0.609710	0.120630	Uiso	1.00	O
O054	1.0	0.240665	0.866441	0.250478	Uiso	1.00	O
O055	1.0	0.379240	0.751120	0.036820	Uiso	1.00	O
O056	1.0	0.628290	0.500930	0.155880	Uiso	1.00	O
O057	1.0	0.877170	0.749973	0.287846	Uiso	1.00	O
O058	1.0	0.236140	0.892490	0.001580	Uiso	1.00	O
O059	1.0	0.485190	0.642300	0.120640	Uiso	1.00	O
O060	1.0	0.739205	0.884662	0.248240	Uiso	1.00	O
O061	1.0	0.522460	0.612150	0.002360	Uiso	1.00	O
O062	1.0	0.011747	0.615040	0.247914	Uiso	1.00	O
O063	1.0	0.771500	0.861960	0.121420	Uiso	1.00	O
O064	1.0	0.017530	0.607210	0.000000	Uiso	1.00	O
O065	1.0	0.266580	0.857010	0.119060	Uiso	1.00	O
O066	1.0	0.514201	0.613998	0.249526	Uiso	1.00	O
O067	1.0	0.233660	0.640080	0.103330	Uiso	1.00	O
O068	1.0	0.486292	0.888802	0.221058	Uiso	1.00	O
O069	1.0	0.723748	0.631899	0.339773	Uiso	1.00	O
O070	1.0	0.738590	0.645020	0.105680	Uiso	1.00	O
O071	1.0	0.210878	0.626624	0.334104	Uiso	1.00	O
O072	1.0	0.986240	0.887786	0.220806	Uiso	1.00	O
O073	1.0	0.228698	0.674778	0.454223	Uiso	1.00	O
O074	1.0	0.448500	0.680317	0.479163	Uiso	1.00	O
O075	1.0	0.310330	0.570980	0.526878	Uiso	1.00	O
O076	1.0	0.380296	0.550562	0.425565	Uiso	1.00	O
O077	1.0	0.471816	0.596287	0.388109	Uiso	1.00	O
La001	1.0	0.134220	0.259760	0.055790	Uiso	1.00	La
La002	1.0	0.382864	0.009985	0.177208	Uiso	1.00	La
La003	1.0	0.625859	0.256300	0.299077	Uiso	1.00	La
La004	1.0	0.619450	0.244940	0.048720	Uiso	1.00	La
La005	1.0	0.119707	0.246807	0.292739	Uiso	1.00	La
La006	1.0	0.868490	0.494740	0.167790	Uiso	1.00	La
La007	1.0	0.621900	0.492480	0.049900	Uiso	1.00	La
La008	1.0	0.870940	0.242280	0.168960	Uiso	1.00	La

La009	1.0	0.128840	0.494557	0.295790	Uiso	1.00	La
La010	1.0	0.136670	0.007290	0.056960	Uiso	1.00	La
La011	1.0	0.384356	0.259100	0.180672	Uiso	1.00	La
La012	1.0	0.630730	0.001569	0.299406	Uiso	1.00	La
La013	1.0	0.134220	0.759760	0.055790	Uiso	1.00	La
La014	1.0	0.384882	0.507357	0.180791	Uiso	1.00	La
La015	1.0	0.628711	0.750772	0.296854	Uiso	1.00	La
La016	1.0	0.619450	0.744940	0.048720	Uiso	1.00	La
La017	1.0	0.118198	0.745868	0.293964	Uiso	1.00	La
La018	1.0	0.868490	0.994740	0.167790	Uiso	1.00	La
La019	1.0	0.621900	0.992480	0.049900	Uiso	1.00	La
La020	1.0	0.870940	0.742280	0.168960	Uiso	1.00	La
La021	1.0	0.124251	0.995895	0.295748	Uiso	1.00	La
La022	1.0	0.136670	0.507290	0.056960	Uiso	1.00	La
La023	1.0	0.382272	0.755145	0.177853	Uiso	1.00	La
La024	1.0	0.618411	0.499078	0.311142	Uiso	1.00	La
Fe001	1.0	0.378060	0.126120	0.052840	Uiso	1.00	Fe
Fe002	1.0	0.631790	0.375319	0.174784	Uiso	1.00	Fe
Fe003	1.0	0.860803	0.123397	0.294201	Uiso	1.00	Fe
Fe004	1.0	0.378060	0.376120	0.052840	Uiso	1.00	Fe
Fe005	1.0	0.622884	0.126710	0.173462	Uiso	1.00	Fe
Fe006	1.0	0.889016	0.378210	0.294010	Uiso	1.00	Fe
Fe007	1.0	0.123055	0.124723	0.173933	Uiso	1.00	Fe
Fe008	1.0	0.878060	0.376120	0.052840	Uiso	1.00	Fe
Fe009	1.0	0.878060	0.126120	0.052840	Uiso	1.00	Fe
Fe010	1.0	0.129254	0.374946	0.174030	Uiso	1.00	Fe
Fe011	1.0	0.358990	0.122691	0.296608	Uiso	1.00	Fe
Fe012	1.0	0.378060	0.626120	0.052840	Uiso	1.00	Fe
Fe013	1.0	0.629704	0.874617	0.173785	Uiso	1.00	Fe
Fe014	1.0	0.861846	0.620837	0.294029	Uiso	1.00	Fe
Fe015	1.0	0.378060	0.876120	0.052840	Uiso	1.00	Fe
Fe016	1.0	0.623567	0.625932	0.174173	Uiso	1.00	Fe
Fe017	1.0	0.889968	0.877119	0.293790	Uiso	1.00	Fe
Fe018	1.0	0.124341	0.624420	0.174544	Uiso	1.00	Fe
Fe019	1.0	0.878060	0.876120	0.052840	Uiso	1.00	Fe
Fe020	1.0	0.391622	0.874139	0.296574	Uiso	1.00	Fe
Fe021	1.0	0.878060	0.626120	0.052840	Uiso	1.00	Fe
Fe022	1.0	0.129803	0.875384	0.174684	Uiso	1.00	Fe
Fe023	1.0	0.363362	0.623750	0.299741	Uiso	1.00	Fe
Cu001	1.0	0.385371	0.371548	0.304606	Uiso	1.00	Cu
S001	1.0	0.333110	0.628619	0.478293	Uiso	1.00	S
H001	1.0	0.493460	0.642599	0.420446	Uiso	1.00	H

4. LFO

```
data_created_by_vaspkit_code
_audit_creation_date         Mon Feb 13 20:23:00 2023
_pd_phase_name                    'CIF files'
_cell_length_a                   10.88689995
_cell_length_b                   15.36690044
_cell_length_c                   22.85919952
_cell_angle_alpha                    90.0000
_cell_angle_beta                     90.0000
_cell_angle_gamma                    90.0632
_symmetry_space_group_name_H-M          'P 1'
_symmetry_Int_Tables_number           1
loop_
_symmetry_equiv_pos_as_xyz
    'x, y, z'
loop_
   _atom_site_label
   _atom_site_occupancy
   _atom_site_fract_x
   _atom_site_fract_y
   _atom_site_fract_z
   _atom_site_thermal_displace_type
   _atom_site_U_iso_or_equiv
   _atom_site_type_symbol
    O001  1.0    0.844420    0.001100    0.053340  Uiso   1.00   O
    O002  1.0    0.100208    0.250015    0.173765  Uiso   1.00   O
    O003  1.0    0.361056    0.998605    0.300035  Uiso   1.00   O
    O004  1.0    0.020010    0.392320    0.104120  Uiso   1.00   O
    O005  1.0    0.267471    0.136784    0.220882  Uiso   1.00   O
    O006  1.0    0.534358    0.375812    0.340613  Uiso   1.00   O
    O007  1.0    0.376880    0.001110    0.068860  Uiso   1.00   O
    O008  1.0    0.626268    0.250846    0.188644  Uiso   1.00   O
    O009  1.0    0.874085    0.999676    0.307418  Uiso   1.00   O
    O010  1.0    0.519980    0.359740    0.104100  Uiso   1.00   O
    O011  1.0    0.765768    0.112203    0.220458  Uiso   1.00   O
    O012  1.0    0.031727    0.369834    0.337517  Uiso   1.00   O
    O013  1.0    0.155721    0.000279    0.175325  Uiso   1.00   O
    O014  1.0    0.911700    0.251140    0.052340  Uiso   1.00   O
    O015  1.0    0.394902    0.247196    0.296032  Uiso   1.00   O
    O016  1.0    0.736100    0.359910    0.001560  Uiso   1.00   O
    O017  1.0    0.985150    0.109710    0.120630  Uiso   1.00   O
    O018  1.0    0.239660    0.362642    0.249738  Uiso   1.00   O
    O019  1.0    0.379240    0.251120    0.036820  Uiso   1.00   O
    O020  1.0    0.628290    0.000930    0.155880  Uiso   1.00   O
    O021  1.0    0.874141    0.250393    0.287720  Uiso   1.00   O
    O022  1.0    0.236140    0.392490    0.001580  Uiso   1.00   O
    O023  1.0    0.485190    0.142300    0.120640  Uiso   1.00   O
    O024  1.0    0.736426    0.388496    0.251302  Uiso   1.00   O
    O025  1.0    0.522460    0.112150    0.002360  Uiso   1.00   O
    O026  1.0    0.011327    0.115752    0.248519  Uiso   1.00   O
    O027  1.0    0.771500    0.361960    0.121420  Uiso   1.00   O
    O028  1.0    0.017530    0.107210    0.000000  Uiso   1.00   O
    O029  1.0    0.266580    0.357010    0.119060  Uiso   1.00   O
    O030  1.0    0.511381    0.111633    0.247652  Uiso   1.00   O
    O031  1.0    0.233660    0.140080    0.103330  Uiso   1.00   O
```

O032	1.0	0.485762	0.386716	0.221061	Uiso	1.00	O
O033	1.0	0.717730	0.130040	0.339208	Uiso	1.00	O
O034	1.0	0.738590	0.145020	0.105680	Uiso	1.00	O
O035	1.0	0.213709	0.128424	0.337641	Uiso	1.00	O
O036	1.0	0.984324	0.387302	0.220570	Uiso	1.00	O
O037	1.0	0.844420	0.501100	0.053340	Uiso	1.00	O
O038	1.0	0.101059	0.750495	0.174393	Uiso	1.00	O
O039	1.0	0.360340	0.495677	0.295606	Uiso	1.00	O
O040	1.0	0.020010	0.892320	0.104120	Uiso	1.00	O
O041	1.0	0.270128	0.636843	0.220606	Uiso	1.00	O
O042	1.0	0.538588	0.878550	0.339552	Uiso	1.00	O
O043	1.0	0.376880	0.501110	0.068860	Uiso	1.00	O
O044	1.0	0.624372	0.750344	0.188510	Uiso	1.00	O
O045	1.0	0.875017	0.501210	0.308624	Uiso	1.00	O
O046	1.0	0.519980	0.859740	0.104100	Uiso	1.00	O
O047	1.0	0.767379	0.613761	0.220341	Uiso	1.00	O
O048	1.0	0.035432	0.871405	0.337389	Uiso	1.00	O
O049	1.0	0.156183	0.499725	0.175923	Uiso	1.00	O
O050	1.0	0.911700	0.751140	0.052340	Uiso	1.00	O
O051	1.0	0.393498	0.749818	0.295300	Uiso	1.00	O
O052	1.0	0.736100	0.859910	0.001560	Uiso	1.00	O
O053	1.0	0.985150	0.609710	0.120630	Uiso	1.00	O
O054	1.0	0.242050	0.866700	0.249589	Uiso	1.00	O
O055	1.0	0.379240	0.751120	0.036820	Uiso	1.00	O
O056	1.0	0.628290	0.500930	0.155880	Uiso	1.00	O
O057	1.0	0.877714	0.750543	0.287472	Uiso	1.00	O
O058	1.0	0.236140	0.892490	0.001580	Uiso	1.00	O
O059	1.0	0.485190	0.642300	0.120640	Uiso	1.00	O
O060	1.0	0.740015	0.885089	0.248104	Uiso	1.00	O
O061	1.0	0.522460	0.612150	0.002360	Uiso	1.00	O
O062	1.0	0.012279	0.615279	0.248099	Uiso	1.00	O
O063	1.0	0.771500	0.861960	0.121420	Uiso	1.00	O
O064	1.0	0.017530	0.607210	0.000000	Uiso	1.00	O
O065	1.0	0.266580	0.857010	0.119060	Uiso	1.00	O
O066	1.0	0.515544	0.611946	0.250505	Uiso	1.00	O
O067	1.0	0.233660	0.640080	0.103330	Uiso	1.00	O
O068	1.0	0.487765	0.889471	0.221348	Uiso	1.00	O
O069	1.0	0.723918	0.633617	0.340466	Uiso	1.00	O
O070	1.0	0.738590	0.645020	0.105680	Uiso	1.00	O
O071	1.0	0.211502	0.625572	0.333938	Uiso	1.00	O
O072	1.0	0.986992	0.888708	0.220749	Uiso	1.00	O
O073	1.0	0.221641	0.671217	0.453698	Uiso	1.00	O
O074	1.0	0.439870	0.685754	0.479314	Uiso	1.00	O
O075	1.0	0.309559	0.571235	0.526869	Uiso	1.00	O
O076	1.0	0.382954	0.552353	0.426472	Uiso	1.00	O
O077	1.0	0.471418	0.600319	0.388480	Uiso	1.00	O
La001	1.0	0.134220	0.259760	0.055790	Uiso	1.00	La
La002	1.0	0.383084	0.009035	0.177202	Uiso	1.00	La
La003	1.0	0.628891	0.252823	0.296694	Uiso	1.00	La
La004	1.0	0.619450	0.244940	0.048720	Uiso	1.00	La
La005	1.0	0.116821	0.245872	0.292783	Uiso	1.00	La
La006	1.0	0.868490	0.494740	0.167790	Uiso	1.00	La
La007	1.0	0.621900	0.492480	0.049900	Uiso	1.00	La
La008	1.0	0.870940	0.242280	0.168960	Uiso	1.00	La

La009	1.0	0.126336	0.495426	0.296267	Uiso	1.00	La
La010	1.0	0.136670	0.007290	0.056960	Uiso	1.00	La
La011	1.0	0.382320	0.256273	0.177186	Uiso	1.00	La
La012	1.0	0.631625	0.001294	0.300044	Uiso	1.00	La
La013	1.0	0.134220	0.759760	0.055790	Uiso	1.00	La
La014	1.0	0.384434	0.509915	0.178677	Uiso	1.00	La
La015	1.0	0.629194	0.752578	0.296776	Uiso	1.00	La
La016	1.0	0.619450	0.744940	0.048720	Uiso	1.00	La
La017	1.0	0.120854	0.745957	0.293861	Uiso	1.00	La
La018	1.0	0.868490	0.994740	0.167790	Uiso	1.00	La
La019	1.0	0.621900	0.992480	0.049900	Uiso	1.00	La
La020	1.0	0.870940	0.742280	0.168960	Uiso	1.00	La
La021	1.0	0.126098	0.995817	0.295831	Uiso	1.00	La
La022	1.0	0.136670	0.507290	0.056960	Uiso	1.00	La
La023	1.0	0.382291	0.756008	0.177950	Uiso	1.00	La
La024	1.0	0.626615	0.504347	0.309719	Uiso	1.00	La
Fe001	1.0	0.378060	0.126120	0.052840	Uiso	1.00	Fe
Fe002	1.0	0.630380	0.375424	0.174655	Uiso	1.00	Fe
Fe003	1.0	0.861488	0.122461	0.294714	Uiso	1.00	Fe
Fe004	1.0	0.378060	0.376120	0.052840	Uiso	1.00	Fe
Fe005	1.0	0.623374	0.126342	0.173598	Uiso	1.00	Fe
Fe006	1.0	0.888219	0.378559	0.294427	Uiso	1.00	Fe
Fe007	1.0	0.123290	0.124454	0.174289	Uiso	1.00	Fe
Fe008	1.0	0.878060	0.376120	0.052840	Uiso	1.00	Fe
Fe009	1.0	0.390783	0.372987	0.296167	Uiso	1.00	Fe
Fe010	1.0	0.878060	0.126120	0.052840	Uiso	1.00	Fe
Fe011	1.0	0.129089	0.375276	0.174685	Uiso	1.00	Fe
Fe012	1.0	0.360291	0.123780	0.296321	Uiso	1.00	Fe
Fe013	1.0	0.378060	0.626120	0.052840	Uiso	1.00	Fe
Fe014	1.0	0.630382	0.874709	0.173720	Uiso	1.00	Fe
Fe015	1.0	0.863501	0.622655	0.294303	Uiso	1.00	Fe
Fe016	1.0	0.378060	0.876120	0.052840	Uiso	1.00	Fe
Fe017	1.0	0.624044	0.625894	0.174022	Uiso	1.00	Fe
Fe018	1.0	0.891261	0.877324	0.293929	Uiso	1.00	Fe
Fe019	1.0	0.124593	0.624514	0.174707	Uiso	1.00	Fe
Fe020	1.0	0.878060	0.876120	0.052840	Uiso	1.00	Fe
Fe021	1.0	0.392296	0.874712	0.296826	Uiso	1.00	Fe
Fe022	1.0	0.878060	0.626120	0.052840	Uiso	1.00	Fe
Fe023	1.0	0.129902	0.875537	0.174630	Uiso	1.00	Fe
Fe024	1.0	0.365053	0.623194	0.300348	Uiso	1.00	Fe
S001	1.0	0.329004	0.629407	0.478254	Uiso	1.00	S
H001	1.0	0.490036	0.648140	0.419947	Uiso	1.00	H

5. LFO-121-PMS

```
data_created_by_vaspkit_code
_audit_creation_date         Mon Feb 13 20:22:29 2023
_pd_phase_name                    'CIF files'
_cell_length_a                   10.88689995
_cell_length_b                   15.36690044
_cell_length_c                   22.85919952
_cell_angle_alpha                    90.0000
_cell_angle_beta                     90.0000
_cell_angle_gamma                    90.0632
_symmetry_space_group_name_H-M           'P 1'
_symmetry_Int_Tables_number           1
loop_
_symmetry_equiv_pos_as_xyz
    'x, y, z'
loop_
   _atom_site_label
   _atom_site_occupancy
   _atom_site_fract_x
   _atom_site_fract_y
   _atom_site_fract_z
   _atom_site_thermal_displace_type
   _atom_site_U_iso_or_equiv
   _atom_site_type_symbol
    O001  1.0    0.844420    0.001100    0.053340  Uiso   1.00   O
    O002  1.0    0.100171    0.250429    0.174211  Uiso   1.00   O
    O003  1.0    0.360981   -0.001090    0.299881  Uiso   1.00   O
    O004  1.0    0.020010    0.392320    0.104120  Uiso   1.00   O
    O005  1.0    0.268107    0.138047    0.220770  Uiso   1.00   O
    O006  1.0    0.540051    0.379290    0.340145  Uiso   1.00   O
    O007  1.0    0.376880    0.001110    0.068860  Uiso   1.00   O
    O008  1.0    0.625629    0.251120    0.188794  Uiso   1.00   O
    O009  1.0    0.875111    0.000991    0.309438  Uiso   1.00   O
    O010  1.0    0.519980    0.359740    0.104100  Uiso   1.00   O
    O011  1.0    0.765844    0.112303    0.220949  Uiso   1.00   O
    O012  1.0    0.037216    0.371203    0.337923  Uiso   1.00   O
    O013  1.0    0.156114    0.000784    0.175836  Uiso   1.00   O
    O014  1.0    0.911700    0.251140    0.052340  Uiso   1.00   O
    O015  1.0    0.395634    0.249101    0.297024  Uiso   1.00   O
    O016  1.0    0.736100    0.359910    0.001560  Uiso   1.00   O
    O017  1.0    0.985150    0.109710    0.120630  Uiso   1.00   O
    O018  1.0    0.243794    0.365785    0.248631  Uiso   1.00   O
    O019  1.0    0.379240    0.251120    0.036820  Uiso   1.00   O
    O020  1.0    0.628290    0.000930    0.155880  Uiso   1.00   O
    O021  1.0    0.875635    0.251090    0.288828  Uiso   1.00   O
    O022  1.0    0.236140    0.392490    0.001580  Uiso   1.00   O
    O023  1.0    0.485190    0.142300    0.120640  Uiso   1.00   O
    O024  1.0    0.741704    0.387873    0.247890  Uiso   1.00   O
    O025  1.0    0.522460    0.112150    0.002360  Uiso   1.00   O
    O026  1.0    0.010847    0.116234    0.248394  Uiso   1.00   O
    O027  1.0    0.771500    0.361960    0.121420  Uiso   1.00   O
    O028  1.0    0.017530    0.107210    0.000000  Uiso   1.00   O
    O029  1.0    0.266580    0.357010    0.119060  Uiso   1.00   O
    O030  1.0    0.511368    0.113003    0.247799  Uiso   1.00   O
    O031  1.0    0.233660    0.140080    0.103330  Uiso   1.00   O
```

O032	1.0	0.489013	0.389794	0.221708	Uiso	1.00	O
O033	1.0	0.717002	0.131045	0.340255	Uiso	1.00	O
O034	1.0	0.738590	0.145020	0.105680	Uiso	1.00	O
O035	1.0	0.213312	0.129160	0.337528	Uiso	1.00	O
O036	1.0	0.987344	0.388440	0.221217	Uiso	1.00	O
O037	1.0	0.844420	0.501100	0.053340	Uiso	1.00	O
O038	1.0	0.100171	0.750429	0.174211	Uiso	1.00	O
O039	1.0	0.360981	0.498910	0.299881	Uiso	1.00	O
O040	1.0	0.020010	0.892320	0.104120	Uiso	1.00	O
O041	1.0	0.268107	0.638047	0.220770	Uiso	1.00	O
O042	1.0	0.540051	0.879290	0.340145	Uiso	1.00	O
O043	1.0	0.376880	0.501110	0.068860	Uiso	1.00	O
O044	1.0	0.625629	0.751120	0.188794	Uiso	1.00	O
O045	1.0	0.875111	0.500991	0.309438	Uiso	1.00	O
O046	1.0	0.519980	0.859740	0.104100	Uiso	1.00	O
O047	1.0	0.765844	0.612303	0.220949	Uiso	1.00	O
O048	1.0	0.037216	0.871203	0.337923	Uiso	1.00	O
O049	1.0	0.156114	0.500784	0.175836	Uiso	1.00	O
O050	1.0	0.911700	0.751140	0.052340	Uiso	1.00	O
O051	1.0	0.395634	0.749101	0.297024	Uiso	1.00	O
O052	1.0	0.736100	0.859910	0.001560	Uiso	1.00	O
O053	1.0	0.985150	0.609710	0.120630	Uiso	1.00	O
O054	1.0	0.243794	0.865785	0.248631	Uiso	1.00	O
O055	1.0	0.379240	0.751120	0.036820	Uiso	1.00	O
O056	1.0	0.628290	0.500930	0.155880	Uiso	1.00	O
O057	1.0	0.875635	0.751090	0.288828	Uiso	1.00	O
O058	1.0	0.236140	0.892490	0.001580	Uiso	1.00	O
O059	1.0	0.485190	0.642300	0.120640	Uiso	1.00	O
O060	1.0	0.741704	0.887873	0.247890	Uiso	1.00	O
O061	1.0	0.522460	0.612150	0.002360	Uiso	1.00	O
O062	1.0	0.010847	0.616234	0.248394	Uiso	1.00	O
O063	1.0	0.771500	0.861960	0.121420	Uiso	1.00	O
O064	1.0	0.017530	0.607210	0.000000	Uiso	1.00	O
O065	1.0	0.266580	0.857010	0.119060	Uiso	1.00	O
O066	1.0	0.511368	0.613003	0.247799	Uiso	1.00	O
O067	1.0	0.233660	0.640080	0.103330	Uiso	1.00	O
O068	1.0	0.489013	0.889794	0.221708	Uiso	1.00	O
O069	1.0	0.717002	0.631045	0.340255	Uiso	1.00	O
O070	1.0	0.738590	0.645020	0.105680	Uiso	1.00	O
O071	1.0	0.213312	0.629160	0.337528	Uiso	1.00	O
O072	1.0	0.987344	0.888440	0.221217	Uiso	1.00	O
La001	1.0	0.134220	0.259760	0.055790	Uiso	1.00	La
La002	1.0	0.383365	0.009752	0.177308	Uiso	1.00	La
La003	1.0	0.629776	0.253343	0.296706	Uiso	1.00	La
La004	1.0	0.619450	0.244940	0.048720	Uiso	1.00	La
La005	1.0	0.119069	0.247078	0.291799	Uiso	1.00	La
La006	1.0	0.868490	0.494740	0.167790	Uiso	1.00	La
La007	1.0	0.621900	0.492480	0.049900	Uiso	1.00	La
La008	1.0	0.870940	0.242280	0.168960	Uiso	1.00	La
La009	1.0	0.126299	0.496379	0.295040	Uiso	1.00	La
La010	1.0	0.136670	0.007290	0.056960	Uiso	1.00	La
La011	1.0	0.383758	0.257064	0.177648	Uiso	1.00	La
La012	1.0	0.632119	0.002450	0.299943	Uiso	1.00	La
La013	1.0	0.134220	0.759760	0.055790	Uiso	1.00	La

La014	1.0	0.383365	0.509752	0.177308	Uiso	1.00	La
La015	1.0	0.629776	0.753343	0.296706	Uiso	1.00	La
La016	1.0	0.619450	0.744940	0.048720	Uiso	1.00	La
La017	1.0	0.119069	0.747078	0.291799	Uiso	1.00	La
La018	1.0	0.868490	0.994740	0.167790	Uiso	1.00	La
La019	1.0	0.621900	0.992480	0.049900	Uiso	1.00	La
La020	1.0	0.870940	0.742280	0.168960	Uiso	1.00	La
La021	1.0	0.126299	0.996379	0.295040	Uiso	1.00	La
La022	1.0	0.136670	0.507290	0.056960	Uiso	1.00	La
La023	1.0	0.383758	0.757064	0.177648	Uiso	1.00	La
La024	1.0	0.632119	0.502450	0.299943	Uiso	1.00	La
Fe001	1.0	0.378060	0.126120	0.052840	Uiso	1.00	Fe
Fe002	1.0	0.631038	0.375352	0.173855	Uiso	1.00	Fe
Fe003	1.0	0.862048	0.123327	0.295490	Uiso	1.00	Fe
Fe004	1.0	0.378060	0.376120	0.052840	Uiso	1.00	Fe
Fe005	1.0	0.623431	0.126453	0.173636	Uiso	1.00	Fe
Fe006	1.0	0.891417	0.378813	0.294726	Uiso	1.00	Fe
Fe007	1.0	0.123453	0.125007	0.174338	Uiso	1.00	Fe
Fe008	1.0	0.878060	0.376120	0.052840	Uiso	1.00	Fe
Fe009	1.0	0.393129	0.375147	0.296939	Uiso	1.00	Fe
Fe010	1.0	0.878060	0.126120	0.052840	Uiso	1.00	Fe
Fe011	1.0	0.130067	0.375798	0.174536	Uiso	1.00	Fe
Fe012	1.0	0.360844	0.124776	0.296674	Uiso	1.00	Fe
Fe013	1.0	0.378060	0.626120	0.052840	Uiso	1.00	Fe
Fe014	1.0	0.631038	0.875352	0.173855	Uiso	1.00	Fe
Fe015	1.0	0.862048	0.623328	0.295490	Uiso	1.00	Fe
Fe016	1.0	0.378060	0.876120	0.052840	Uiso	1.00	Fe
Fe017	1.0	0.623431	0.626453	0.173636	Uiso	1.00	Fe
Fe018	1.0	0.891417	0.878813	0.294726	Uiso	1.00	Fe
Fe019	1.0	0.123453	0.625007	0.174338	Uiso	1.00	Fe
Fe020	1.0	0.878060	0.876120	0.052840	Uiso	1.00	Fe
Fe021	1.0	0.393129	0.875147	0.296939	Uiso	1.00	Fe
Fe022	1.0	0.878060	0.626120	0.052840	Uiso	1.00	Fe
Fe023	1.0	0.130067	0.875798	0.174536	Uiso	1.00	Fe
Fe024	1.0	0.360844	0.624776	0.296674	Uiso	1.00	Fe

6. LFO-121-surface

```
data_created_by_vaspkit_code
_audit_creation_date         Thu Feb  9 21:02:29 2023
_pd_phase_name                    'CIF files'
_cell_length_a                    5.42878591
_cell_length_b                    7.71284919
_cell_length_c                    5.43728328
_cell_angle_alpha                    90.0000
_cell_angle_beta                     90.0000
_cell_angle_gamma                    90.0000
_symmetry_space_group_name_H-M          'P 1'
_symmetry_Int_Tables_number          1
loop_
_symmetry_equiv_pos_as_xyz
    'x, y, z'
loop_
   _atom_site_label
   _atom_site_occupancy
   _atom_site_fract_x
   _atom_site_fract_y
   _atom_site_fract_z
   _atom_site_thermal_displace_type
   _atom_site_U_iso_or_equiv
   _atom_site_type_symbol
    O001  1.0    0.717374    0.534693    0.217452  Uiso   1.00   O
    O002  1.0    0.282626    0.465307    0.782548  Uiso   1.00   O
    O003  1.0    0.782626    0.465307    0.717452  Uiso   1.00   O
    O004  1.0    0.217374    0.534693    0.282548  Uiso   1.00   O
    O005  1.0    0.282626    0.034693    0.782548  Uiso   1.00   O
    O006  1.0    0.717374    0.965307    0.217452  Uiso   1.00   O
    O007  1.0    0.217374    0.965307    0.282548  Uiso   1.00   O
    O008  1.0    0.782626    0.034693    0.717452  Uiso   1.00   O
    O009  1.0    0.509890    0.750000    0.566080  Uiso   1.00   O
    O010  1.0    0.490110    0.250000    0.433920  Uiso   1.00   O
    O011  1.0    0.990110    0.250000    0.066080  Uiso   1.00   O
    O012  1.0    0.009890    0.750000    0.933920  Uiso   1.00   O
   La001  1.0    0.529660    0.250000    0.995079  Uiso   1.00  La
   La002  1.0    0.470340    0.750000    0.004921  Uiso   1.00  La
   La003  1.0    0.970340    0.750000    0.495079  Uiso   1.00  La
   La004  1.0    0.029660    0.250000    0.504921  Uiso   1.00  La
   Fe001  1.0    0.500000    0.000000    0.500000  Uiso   1.00  Fe
   Fe002  1.0    0.000000    0.000000    0.000000  Uiso   1.00  Fe
   Fe003  1.0    0.500000    0.500000    0.500000  Uiso   1.00  Fe
   Fe004  1.0    0.000000    0.500000    0.000000  Uiso   1.00  Fe
```
